# Physicochemical, nutritional, and quality parameters of salted semidried mullet (*Chelon haematocheilus*) prepared with different processing methods

**DOI:** 10.1002/fsn3.1270

**Published:** 2019-11-16

**Authors:** Hee Geun Jo, Min Ji Kim, Bo Yeong Moon, Yong Sik Sin, Kyoung Seon Lee, Sun Hee Cheong

**Affiliations:** ^1^ Department of Marine Bio Food Science College of Fisheries and Ocean Science Chonnam National University Yeosu Korea; ^2^ Department of Environmental Engineering & Biotechnology Mokpo National Maritime University Mokpo Korea

**Keywords:** *Chelon haematocheilus*, nutritional characteristics, physicochemical, salted semidried mullet, sanitary quality

## Abstract

The mullet (*Chelon haematocheilus*) is a cosmopolitan coastal species. It is often consumed as a sliced raw fish in Korea and as a dried and salted fish roe in several countries, including the southeastern United States and Japan. In this study, to optimize traditional processing of salted semidried mullet (SSDM) for the development of high‐quality products, nine different types of traditional process were applied, and quality changes including physicochemical, nutritional, and sanitary properties were observed. The approximate composition of SSDM was as follows: moisture, 66.1% to 71.8%; ash, 1.65% to 3.75%; crude protein, 16.12% to 18.09%; and crude lipid, 1.11% to 2.07%. The salinity, water activity (*Aw*), color parameters, peroxide value (POV), acid value (AV), thiobarbituric acid (TBA), and the total volatile basic nitrogen (TVB‐N) contents in fresh mullet (FM) and different SSDM groups were affected by different processing techniques including salt concentration and drying methods. In particular, the salinity was significantly increased, whereas the *Aw* was significantly decreased in all SSDM groups compared to those of FM group. In both FM and SSDM groups, the AV, POV, and TBA values gradually increased with prolonged storage and crude fat content; however, they were not affected by salinity. The amino and fatty acid content also varied depending on the processing method; however, the composition and protein patterns were similar among the groups. The total aerobic bacterial numbers of all SSDM groups were also influenced by different processing methods. The microbial numbers in the mullet after salted semidried treatment were markedly lower than in the FM group during refrigerated storage for 14 days. Therefore, salted semidried treatment for mullet show extended shelf life and improved microbiological safety and biochemical parameters during refrigerated storage.

## Graphical abstract

Salted semidried mullet show extended shelf life and improved microbiological safety and biochemical parameters during refrigerated storage.

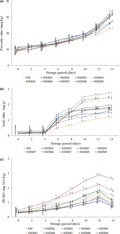

## 1 INTRODUCTION

Fish and shellfish are not only sources of highly unsaturated fatty acids including eicosapentaenoic acid (EPA) and docosahexaenoic acid (DHA) but also sources of animal protein containing a large amount of valuable nutritional components including vitamins and minerals (Ali et al., 2019). Fish and shellfish consumption has been linked to increased risk of blood cholesterol levels, cardiovascular diseases such as atherosclerosis and hypertension, and several forms of cancers (Lauzon et al., 2010). However, many researchers have demonstrated that nutrients and other bioactive components, such as n‐3 PUFAs, protein, fiber, taurine, sterol, and pigments derived from fish and shellfish, have a number of biological activities, including anticoagulant (Matsubara, Matsuura, Hori, & Miyazawa, 2000), antioxidant (Heo, Park, Lee, & Jeon, 2005), anticancer (Bouic, 2001), anti‐inflammatory (Kim, Rajapakse, & Kim, 2009), antihypertensive (Harada et al., 2004), and antihypercholesterolemic effects (Matsushima et al., 2003). On the other hand, it is well known that during storage, due to the high amounts of omega‐3 polyunsaturated fatty acids and moisture, fresh fish and their products are susceptible to peroxidation that modifies both nutritional quality and sensory characteristics (Maqsood, Benjakul, & Shahidi, 2013). Therefore, research efforts have highlighted the possibility to extend the shelf life of fish include freezing, drying, salting, and canning. Freezing methods have been commonly used to extend the storage and distribution of processed fish products; however, if thawed, the fish meat can easily crumble leading to dry texture (Ma, Wu, Zhang, Giovanni, & Meng, 2018). In addition, the drying method, which is a relatively simple and classical method, can impair sensual and physical properties such as fat oxidation, browning, and texture due to excessive drying (Lee, Kim, Chae, & Chang, 2007). Recent trends worldwide suggest a demand for semidried foods or semimoist foods such as squid (Gou, Choi, & Ahn, 2012), horse mackerel (Yang, 1997), mackerel (Song, Lee, Han, Yoon, & Hwang, 2005), brown croaker (Joo, 2011), and salmon (You, 1997) with features very similar with fresh food products, but with a longer shelf life (Qiu, Zhang, Tang, Adhikari, & Cao, 2019).

Mullet (*Mugil cephalus* L.) is a marine fish belonging to the family Mugilidae and lives in tropical, subtropical, and temperate coastal waters of the world's major oceans (Thomson, 1966). Mullet has a relatively high fat composition compared to other fish species (Marais & Erasmus, 1977). In particular, mullet roe is considered a nutritious food, with well‐balanced protein content including essential amino acids and large amounts of ω3 unsaturated fatty acids, such as 20:5ω3 (EPA) and 22:6ω3 (DHA), known to act an important role in the prevention of cardiovascular diseases (Lu, Ma, Williams, & Chung, 1979). Although a few studies analyzing the chemical composition, bioavailability, and quality during storage of mullet or its roe have been conducted (Çelik, Altielataman, Dincer, & Acarli, 2012; Cho, Rhee, & Kim, 1989; Kim, Seong, et al., 2009; Lee & Park, 1985), the nutritional and quality characteristics of salted semidried mullet (SSDM) meats have yet to be investigated. Therefore, the purpose of this study was to provide basic information to establish the scientific processing conditions and extend the shelf life by investigating the physicochemical, nutritional, and quality characteristics of SSDM prepared by different processing methods during refrigerated storage.

## 2 MATERIALS AND METHODS

### 2.1 Sample preparation

Whole fresh mullets (*Chelon haematocheilus*) were obtained from a fish farm in Jeung‐do (Korea). The average body weight and length of fresh mullet were 1.05 ± 0.28 kg and 49.21 ± 2.76 cm, respectively. Blood and other wastes were removed with tap water, and 5 individual fresh mullets per group were selected, and then, the SSDMs were manufactured immediately by nine manufacturers using different salting and drying procedures based on traditional salting and semidry methods and then called “SSDM 1 ~ 9.” As shown in Table 1, SSDM preparation was conducted by using “dry salting” for SSDM1 ~ 6 or “brine salting” for SSDM7 ~ 9 according to the salting and drying conditions in Table 1. As for dry salting, fresh mullets were put in polystyrene boxes with one layer of salt and one layer of mullets for 3 hr 30 min, and then, they were held in a dry cool place (approximately 20°C) to be semidried for 3 days. As for brine salting, fresh mullets were immersed in salt solution for 4 hr 30 min and then semidried for 4 days.

**Table 1:** Pretreatment methods, salting, and drying conditions for the preparation of salted semidried mullet

Groups	Weight (kg)	Length (cm)	Incision site	Washes (time)	Blood removal	Salting method	Salting time	Salt amount (g/kg)	Drying method	Drying height (m)	Drying times (days)
SSDM1	1.45 ± 0.48	51.40 ± 4.72	Back (full incision)	1	Washing	Dry salting	3 hr 30min	11.9 ± 2.8	Individual drying	5	3
SSDM2	1.01 ± 0.14	49.40 ± 4.77	Back (full incision)	1	Washing	Dry salting	3 hr 30 min	23.5 ± 10.4	Individual drying	5	3
SSDM3	1.21 ± 0.19	49.80 ± 0.84	Back (full incision)	1	Washing	Dry salting	3 hr 30 min	13.9 ± 1.5	Individual drying	5	3
SSDM4	1.02 ± 0.09	47.60 ± 2.30	Abdomen (half‐incision)	1	Washing	Dry salting	3 hr 30 min	31.8 ± 7.3	Individual drying	5	3
SSDM5	1.02 ± 0.31	47.88 ± 3.66	Abdomen (half‐incision)	1	Washing	Dry salting	3 hr 30 min	27.8 ± 4.4	Individual drying	5	3
SSDM6	0.91 ± 0.14	49.60 ± 3.29	Abdomen (half‐incision)	1	Washing	Dry salting	3 hr 30 min	28.4 ± 4.0	Individual drying	5	3
SSDM7	1.57 ± 0.50	52.20 ± 3.27	Back (full incision)	2	Dipping	Brine salting	4 hr 30 min	26.7 ± 3.0	Bundle drying	3	4
SSDM8	1.01 ± 0.23	48.80 ± 3.27	Back (full incision)	2	Dipping	Brine salting	4 hr 30 min	37.0 ± 5.3	Bundle drying	3	4
SSDM9	1.02 ± 0.15	49.00 ± 3.08	Back (full incision)	2	Dipping	Brine salting	4 hr 30 min	33.3 ± 1.8	Bundle drying	3	4

Note

Values are means ± *SD* (*n* = 5).

Abbreviation: SSDM, salted semidried mullet.

### 2.2 Proximate composition, salinity, water activity (*Aw*), and chromaticity

The moisture, protein, and ash contents of SSDM samples were determined using methods described by the Association of Official Analytical Chemists (AOAC, 2012). Moisture content was determined by drying the samples at 105°C until constant weight (AOAC method 950.46B). The protein content was determined using Kjeldahl procedure (AOAC method 955.04). The total lipid content was determined by the method of Bligh and Dyer (1959). The ash content was evaluated by sample incineration in a muffle furnace at 550°C (AOAC method 920.153). For salinity measurement, five times (w/v) deionized water was added to the SSDM sample, and the filtrate obtained by stirring and centrifugation was measured with a salinity meter (PAL‐ES, ATAGO). *Aw* was determined by an Electric Hygrometer (Hygrodynamics, Inc.) at 27°C. The chromaticity was measured with a color meter (ZE2000, Nippon Denshoku Co.) using the muscular part of the SSDM as a sample, and the L* value (lightness: L* = 0 for black, L* = 100 for white), a* value (red/green: +a* = redness, −a* = greenness), and b* value (yellow/blue: +b* = yellowness, −b* = blueness) were recorded. The *L* value of the standard white plate of the color difference meter was 97.50; the *a* and *b* values were −0.27 and 0.21, respectively. Each group of samples was measured five times, and the mean values were obtained.

### 2.3 Lipid oxidation

Lipid oxidation of SSDM samples was assessed according to the acid value (AV), peroxide value (PV), and the thiobarbituric acid (TBA) levels. The AV was determined using the procedure described by Pearson (1970). Briefly, 1 g of the extracted oil sample was dissolved in the equal volumes of diethyl ether and ethanol and 1% phenolphthalein solution was added as an indicator and titrated against 0.1 mol/L NaOH. The AV was subsequently calculated. The PVs were determined using the procedure described by Egan, Kirk, and Sawyer (1981). SSDM samples were ground to a powder, and 0.5 g of the sample was mixed with a 25 ml solution of acetic acid and chloroform (3:2, *v*/*v*) and 1 ml of saturated potassium iodide. The mixture was stored in the dark for about 10 min and then added a 30 ml of distilled water and 1 ml of 1% starch (*w*/*v*) solution. The sample was titrated with 0.01 N sodium thiosulfate until the blue color disappeared. The PVs were expressed as milliequivalents of peroxide oxygen per kg of sample (mEq/kg). The lipid peroxidation was evaluated by measuring TBA levels using the modified method of Faustman, Specht, and Malkus (1992). A 20 g of each mullet sample was homogenized with 50 ml of distilled water and then 10 ml of trichloroacetic acid (15%, final concentration) for 15 s, and then, the homogenate was centrifuged at 33,540 *g* force for 5 min. After filtration of the supernatant using Whatman No. 1 filter paper, 2 ml of 0.06 mol/L thiobarbituric acid was added to 8 ml of the filtrate. The mixture was vortexed for 15 s, heated at 95°C for 1 hr, and then cooled on ice. The absorbance was measured at 532 nm using a UV–vis spectrophotometer, and then, the results were expressed as mg malondialdehyde (MDA) equivalent/kg of sample.

### 2.4 Determination of amino nitrogen content

Amino nitrogen content was evaluated using the formol titration method (Northrop, 1926). Briefly, 5 ml of the SSDM sample was diluted up to 250 ml with distilled water. For the first titration, each diluted sample was titrated with 0.01 mol/L NaOH (pH 8.5). 20 ml of formaldehyde solution (pH 8.5) was added to the diluted sample and then titrated with 0.1 mol/L NaOH for the second titration. The volume of base consumed in both first and second titration was used to calculate the amino nitrogen content.

### 2.5 Determination of total volatile basic nitrogen (TVB‐N)

The TVB‐N was determined via the microtitration method described by Gharibzahedi and Mohammadnabi (2017). Briefly, 5.0 g SSDM sample was homogenized with 50 ml of distilled water using a high‐speed homogenizer (IKA‐T25). The mixture was centrifuged at 10,000 *g* and 4°C for 5 min. A 5 ml of the supernatant was added to 5 ml MgO (10 g/L) and then distilled with a Kjeldahl nitrogen apparatus (KN‐520, Alva instrument). The distillate was obtained with 20 ml of boric acid (0.02 g/L) containing methyl red (1 g/L) and methylene blue (1 g/L) in ethanol as a mixed indicator. The mixed solution was titrated using 0.01 mol/L HCl solution, and 5 ml of distilled water was used instead of the sample as a blank test. The TVB‐N value was calculated based on the consumption of HCl according to the following equation:

$$\text{TVB}-N\;\left(\text{mg}/\%\right)=\frac{\left(V_{1}-V_{2}\right)\;\times \;C\;\times \;14}{m\;\times \;5/50}\times 100$$

where *V*_1_ and *V*_2_ are volume (mL) of HCl used for the sample and the blank, respectively. *C* refers to the concentration of HCl (mol/L). *m* indicates the sample weight (g).

### 2.6 Fatty acid analysis

To analyze the fatty acid, total lipids obtained from the SSDM samples were extracted using a mixture of chloroform:methanol (2:1, v:v) including 0.01% butylated hydroxytoluene. The extracted lipids were dried using a rotary evaporator (VV 2011, Heidolph Co., Ltd) in vacuum and then converted to fatty acid methyl esters (FAMEs) through base‐catalyzed transesterification with sodium methoxide for 2 hr at 30°C (Qwele et al., 2013). FAMEs were quantified using gas chromatography (Shimadzu GC‐17A, Shimadzu, Tokyo, Japan) fused with silica capillary column (SP^TM^‐2560, 100 m × 0.25 mm i.d, 0.25‐μm film thickness, Supelco). Fatty acid analysis was carried out using an initial isothermic period of 140°C for 10 min, followed by a temperature increase at the rate of 4°C/min to 240°C and an isothermic period of 240°C for 30 min. FAMEs n‐hexane (1 μl) was injected into the column. The injection and detector port were maintained at 260°C, with helium gas. The compositions of fatty acid were identified by comparing the retention times of FAME peaks with the standard (47885‐U, Supelco 37 Component FAME Mix, Supelco) and then quantified as mg per kg of SSDM samples using the internal standard. The total fatty acid content was expressed as g per 100 g of samples, while individual fatty acid composition was expressed as a weight percentage of the total fatty acids.

### 2.7 Amino acid composition

To analyze the amino acid, 80 mg of SSDM samples was mixed with 10 ml of 6 N HCl solution. After purging with N_2_ gas in a test tube, the samples were hydrolyzed in a dry oven at 110°C for 24 hr. The hydrolyzed samples were evaporated and added a sodium‐distilled buffer (pH 2.2). Samples were filtered using a syringe filter (0.45 μm) and then analyzed amino acids by reaction with ninhydrin using Biochrom 20 amino acid analyzer (Pharmacia Biotech). Amino acid composition was determined by measuring absorbance at 440 and 570 nm, respectively.

### 2.8 Sodium dodecyl sulfate–polyacrylamide gel electrophoresis (SDS‐PAGE)

To analyze the protein profile of the SSDM, 500 μl of lysis buffer was added to 0.1 g of the sample, homogenized, and centrifuged at 12,000 *g* for 30 min to separate the supernatant. Protein in the supernatant was quantified, mixed with sample buffer, then heated at 100°C for 5 min, and used for SDS‐PAGE analysis. Electrophoresis was conducted using a Mini‐PROTEAN Tetra Cell (Bio‐Rad Lab., Inc.) according to the method of Laemmli (1970), and a 10–15 μl sample was injected. SDS‐PAGE was performed for 90 min.

### 2.9 Microbiological analyses

Microbiological analyses were conducted using a commercially available 3M™ Petrifilm™ Plates (3M Microbiology Products), according to the methods suggested by the manufacturer. Briefly, The SSDM samples (10 g) were placed in a sterilized pack (3M^TM^ Sample Bag) and homogenized with 100 ml physiological saline (0.85%) for 2 min. The pretreated samples were cultured in 3M™ Petrifilm™ Plates (3M Microbiology Products) at 35 ± 1°C for 48 hr, and then, the number of red colonies was counted. The average number of colonies was multiplied by the dilution factor. All counts were expressed as log_10_ cfu/g.

### 2.10 Coliforms and *Escherichia coli* (*E. coli*)

Microbiological analyses were conducted using a commercially available 3M™ Petrifilm™ E. coli/Coliform Count Plate (3M Microbiology Products), according to the methods suggested by the manufacturer. SSDM samples (10 g) were placed in a sterilized pack (3M^TM^ Sample Bag) supplemented with 0.9% (*v*/*w*) of 0.85% physiological saline and homogenized for 2 min. The sample suspension (1 ml) was cultured in 3M dry petrical medium (3M Microbiology Products) and incubated at 35 ± 1°C for 24 hr. Red colonies surrounded with trapped gas represented coliforms, whereas blue colonies with trapped gas were identified as *E. coli*. Each dilution was conducted in duplicate, and plates containing 15–150 colonies were recommended for counting. The colony‐forming unit (CFU) per gram of sample was used, and the minimum limit for detection was log cfu/g.

### 2.11 Statistical analysis

All data are expressed as means ± *SD*. Statistical analyses were carried out using IBM SPSS statistic ver. 20. The data were evaluated by one‐way analysis of variance. Differences between mean values were assessed using the Duncan's multiple range test. Differences were considered statistically significant when the *p* value was <.05.

## 3 RESULTS AND DISCUSSION

### 3.1 Proximate composition, salinity, and water activity (*Aw*)

In this study, the approximate composition, salinity, and water activity of SSDM samples were treated with nine different traditional methods, as shown in Table 2. The composition of fish muscle varies depending on species, age, season, diets, stage of maturity, organs, and muscle location (Noël et al., 2011). The content of moisture, protein, fat, and ash in the fish body commonly ranges from 60% to 81%, 16 to 21%, 0.1 to 25%, and 0.4 to 1.5%, respectively (Muraleedharan, Antony, Perigreen, & Gopakumar, 1996). Norouzi and Bagheri (2015) reported that the chemical composition of golden gray mullet during sexual arrest and maturity was as follows: fat, 2.22%–3.94%; protein, 21.81%–22.85%; moisture, 77.39%–78.13%; and ash, 1.35%–1.48%, respectively. According to the literature, the body composition of *M. cephalus* was comprised of 74.5% moisture, 17.5% protein, 2.7% fat, and 4.9% ash (Marais & Erasmus, 1977). Akbary (2019) also reported that the carcass chemical composition of gray mullet was composed of 71.98–74.76 moisture, 17.84–18.82 crude protein, 2.11–5.91 crude lipid, and 5.84–7.14 crude ash, respectively. In the present study, the SSDM samples showed significantly lower moisture and higher protein and ash content compared with those of fresh mullet samples. The crude fat and crude protein contents ranged from 1.11% to 2.07% and from 16.12% to 18.09% in all the SSDM groups. Siriskar, Khedkar, and Lior (2013) demonstrated that the protein and fat content decreased, while the ash content remained constant in salted and pressed anchovies. On the other hand, it has been reported that the dried caviar from flathead gray mullet showed significantly lower moisture and higher protein contents due to drying effect on evaporating water partially out of the product resulting in an increase in dry weight (Çelik et al., 2012). In addition, the higher ash content resulted from moisture loss and concentration of chemical components after the drying process (Akonor, Ofori, Dziedzoave, & Kortei, 2016). In the present study, the salinity of the SSDM groups was significantly higher than that of the FM group due to the salt pretreatment. It has been reported that the salt content of the anchovies ranges from 0.55% to 0.58% which is typical of marine species (Siriskar et al., 2013). Yin, Kim, Noh, and Choi (2013) reported that the salinity of cod bone stock was 0.49%. Similar to our results, it has also been reported that the salinity of mussel stock was 0.71% (You, Shin, Choi, & Seo, 2013). In our study, the salinity was the lowest in the SSDM9 group and the highest in SSDM8 group among the different groups. However, the salinity of all SSDM groups ranged from 1.48% to 3.42% and was lower than the human threshold values of 3.0%, which is chiefly considered as a factor leading to hypertension (Amerine, Panborn, & Roessler, 1965). On the other hand, the *Aw* of the SSDM groups was significantly lower than that of the FM group. The mean *Aw* of the SSDM samples was within 0.92–0.98. These results indicated that the proximate composition, salinity, and *Aw* of SSDM samples were significantly affected by differences in the traditional methods including salting and drying techniques.

**Table 2:** Proximate composition, salinity, and water activity of fresh and salted semidried mullet

Groups	Proximate composition					
	Moisture (%)	Ash (% fw)	Crude fat (% fw)	Crude protein (% fw)	Salinity (%)	Water activity
FM	79.18 ± 1.31^a^	1.45 ± 0.18^c^	1.74 ± 0.04^b^	14.11 ± 0.04^e^	0.74 ± 0.03^f^	0.99 ± 0.001^a^
SSDM1	71.80 ± 1.27^b^	1.65 ± 0.05^c^	1.50 ± 0.18^bc^	16.12 ± 0.25^d^	1.84 ± 0.03^d^	0.98 ± 0.001^b^
SSDM2	71.66 ± 1.30^b^	1.74 ± 0.17^c^	1.63 ± 0.06^bc^	16.74 ± 0.18^cd^	2.50 ± 0.13^b^	0.98 ± 0.001^b^
SSDM3	70.19 ± 0.85^bc^	1.92 ± 0.02^c^	2.07 ± 0.04^a^	17.24 ± 0.18^bc^	2.16 ± 0.10^c^	0.98 ± 0.001^b^
SSDM4	67.56 ± 0.95^de^	3.75 ± 0.07^a^	1.58 ± 0.07^bc^	18.09 ± 0.16^a^	2.10 ± 0.06^cd^	0.94 ± 0.01^d^
SSDM5	66.10 ± 1.48^e^	3.51 ± 0.25^a^	1.46 ± 0.06^c^	16.83 ± 0.26^cd^	2.74 ± 0.03^b^	0.96 ± 0.003^c^
SSDM6	71.36 ± 1.67^b^	3.63 ± 0.21^a^	1.58 ± 0.09^bc^	17.84 ± 0.31^ab^	2.90 ± 0.03^b^	0.94 ± 0.008^cd^
SSDM7	68.82 ± 1.40^cd^	3.52 ± 0.17^a^	1.31 ± 0.07^cd^	17.72 ± 0.56^ab^	2.14 ± 0.06^c^	0.92 ± 0.005^e^
SSDM8	67.05 ± 1.30^de^	2.51 ± 0.28^b^	1.97 ± 0.07^a^	16.66 ± 0.15^cd^	3.42 ± 0.20^a^	0.92 ± 0.004^e^
SSDM9	70.51 ± 1.60^bc^	2.62 ± 0.24^b^	1.11 ± 0.07^d^	16.40 ± 0.45^cd^	1.48 ± 0.03^e^	0.96 ± 0.01^c^

Note

Values represent mean ± standard deviation (*SD*) (*n* = 5). Different superscript letters within each column represent significant differences (*p* < .05).

Abbreviations: FM, fresh mullet; fw, fresh weight; SSDM, salted semidried mullet.

### 3.2 Chromaticity

In the present study, the values of the color parameters are listed in Table 3. The L (lightness) values indicate blackness and whiteness, a value indicates redness and greenness, and b denotes degree of yellowness and blueness. The “*a*” value of the colorimeter was red when the + value was higher, and green when the ‐value was higher. The “*b*” value indicated a yellow color when the + value was displayed, and blue when the value shifted to a negative value. Yellowness (b*) is closely related to lipid oxidation of fish flesh (Hong, Luo, Zhou, & Shen, 2012). In general, color measurement is an important parameter in processed fish products because of consumers’ association with a characteristic of fish and their products (Çelik et al., 2012). In the present study, significant changes occurred (*p* < .05) in the *L*, a*, and b* values of the groups (fresh and SSDM samples) with the different salting or processing methods. In this study, the average values of redness (a*) were in the range of −1.34–2.43, and those of yellowness (b*) were in the range of 6.84–12.27. In particular, *L* and b* values of SSDM samples were significantly increased except in SSDM1 group compared with those of the fresh mullet samples. These results may be attributed to the soaked salting and drying process in SSDM groups. Çelik et al. (2012) also reported that the darkness of dried flathead gray mullet caviar was contributed by the increasing level of a* and b* values.

**Table 3:** Color parameters of fresh and salted semidried mullet

	Color value			
	L*	a*	b*	△*E* Value
Groups				
FM	33.68 ± 0.05^f^	0.22 ± 0.02^c^	7.51 ± 0.03^g^	64.65 ± 0.06^b^
SSDM1	31.10 ± 0.04^g^	−1.34 ± 0.05^e^	6.84 ± 0.07^i^	66.73 ± 0.04^a^
SSDM2	34.71 ± 0.03^e^	0.01 ± 0.27^c^	9.31 ± 0.16^d^	63.43 ± 0.03^c^
SSDM3	40.00 ± 0.07^c^	−0.62 ± 0.02^d^	8.56 ± 0.01^e^	58.09 ± 0.07^e^
SSDM4	50.52 ± 0.12^a^	0.05 ± 0.02^c^	10.50 ± 0.01^b^	48.08 ± 0.11^g^
SSDM5	35.73 ± 0.14^d^	1.53 ± 0.05^b^	10.18 ± 0.09^c^	62.58 ± 0.13^d^
SSDM6	35.41 ± 0.17^d^	−0.38 ± 0.04^d^	8.34 ± 0.08^f^	62.61 ± 0.16^d^
SSDM7	50.66 ± 0.15^a^	2.43 ± 0.02^a^	10.54 ± 0.00^b^	48.03 ± 0.15^g^
SSDM8	44.51 ± 0.13^b^	2.31 ± 0.01^a^	12.27 ± 0.02^a^	54.40 ± 0.13^f^
SSDM9	34.66 ± 0.13^e^	−0.39 ± 0.01^d^	7.04 ± 0.03^h^	63.20 ± 0.12^c^

Note

Values represent mean ± standard deviation (*SD*) (*n* = 5). Different superscript letters within each column represent significant differences (*p* < .05) for *(lightness), for a*(redness), and for b*(yellowness).

Abbreviations: FM, fresh mullet; SSDM, salted semidried mullet.

### 3.3 Changes in peroxide value (POV), acid value (AV), and thiobarbituric acid (TBA) values during refrigerated storage

**Figure 1:**
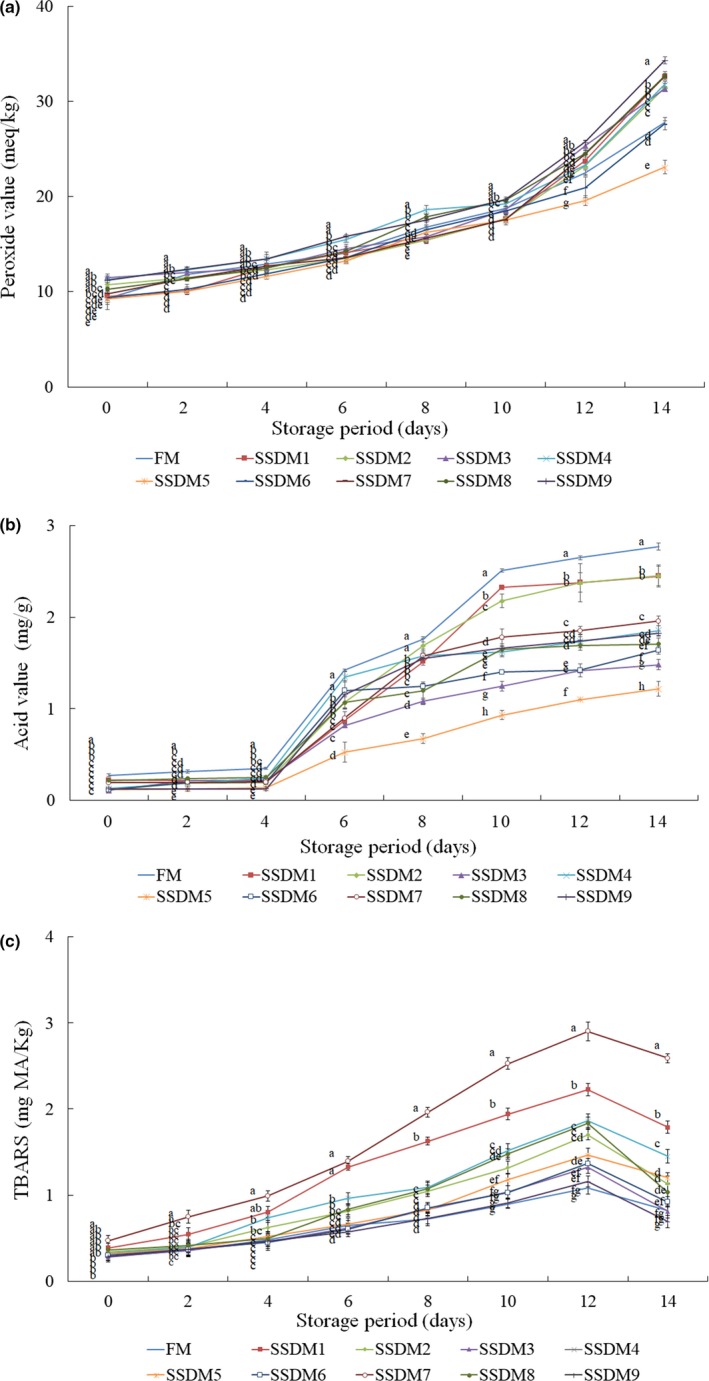
Changes in peroxide value (POV) (a), acid value (AV) (b), and TBA (c) in salted semidried mullet (SSDM) during storage at 4°C for 14 days. Values represent mean ± standard deviation (*SD*) (*n* = 5). Different superscript letters represent significant differences (*p* < .05)

Changes in lipid oxidation indices including POV, AV, and TBA values of SSDM during the 14 days of storage at 4°C are shown in Figure 1. The POV was measured to examine the lipid stability of fresh and SSDM samples during refrigerated storage. The POV is related to rancidity in the early stages of lipid oxidation and is a good indicator of the rate of oxidation (Kim, Kim, Park, Kim, & Lee, 2001). As shown in Figure 1a, the POV gradually increased in all groups with extended storage period. The SSDM3 group showed the greatest increase from 11.04 to 34.75 meq/kg, whereas the SSDM5 group showed the smallest increase from 9.12 to 23.60 meq/kg during refrigerated storage. In general, the AV increases with the deterioration or rancidity of the oil (Falade & Oboh, 2015). In the present study, similar to POV, the AV also gradually increased with increasing storage period in all groups. The AV increased sharply after day 4 of the storage in all groups. In particular, the FM group showed the greatest increase from 0.3 to 2.8 mg/g, whereas the SSDM5 group showed the smallest increase from 0.1 to 1.5 mg/g during refrigerated storage. The lipids in fish are decomposed by air, and lipolytic and lipoxidative enzymes during processing or storage, and these oxidative products may turn increasingly rancid through oxidation (Cai et al., 2014). In general, the level of TBA used to determine the degree of oxidative rancidity of lipids as indicated above should be at least 3 in a very good material and a maximum of 5 in a good material, and the range of acceptability is between 7 and 8 (Taşkaya & Yaşar, 2018). In the present study, all groups showed a graduated increase in TBA depending on the storage period; however, it decreased on days 12 until 14 of storage (Figure 1c). It was found that the SSDM9 group showed the lowest TBA value (0.69 mg MA/kg), whereas the SSDM7 group had the highest TBA value (2.59 mg MA/kg) on day 14 of storage among all groups. Witte, Krause, and Baile (1970) reported that the TBA values increased with storage period, because of carbonyl compounds, alcohols, ketones, aldehydes, and other oxidative and hydrolytic products derived from fats during aging of meat. It has also been reported that the TBA value in mullet roe products was affected by physical state of the matrix, manufacturing procedures, and storage (Rosa et al., 2009). Similarly, Guizani, Rahman, Al‐Ruzeiqi, Al‐Sabahi, and Sureshchandran (2014) demonstrated that POV in hot‐smoked tuna showed an inverse correlation with salt concentration and the values of POV and thiobarbituric acid‐reactive substances (TBARS) increased with storage period.

### 3.4 Amino nitrogen and total volatile basic nitrogen (TVB‐N) values during refrigerated storage

**Figure 2:**
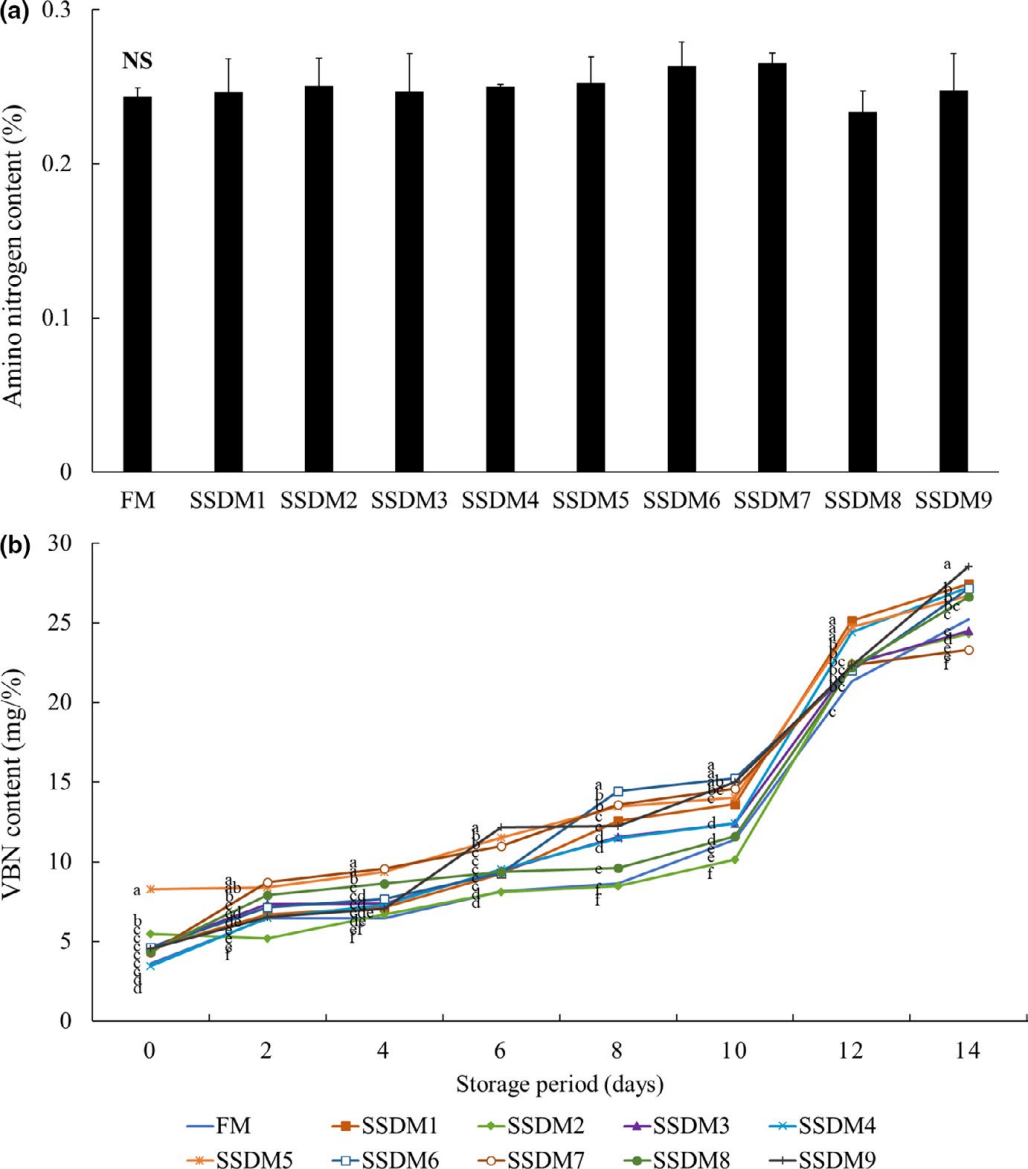
Amino nitrogen content (a) and changes of TVB‐N (b) contents of salted semidried mullet (SSDM) during storage at 4°C for 14 days. Values represent mean ± standard deviation (*SD*) (*n* = 5). Different superscript letters represent significant differences (*p* < .05)

In the present study, the initial values of amino nitrogen content were not significantly different in all groups (Figure 2a). The TVB‐N values are an important parameter for the evaluation of loss of freshness and chemical degradation of fish. The TVB‐N levels of trimethylamine (TMA) and dimethylamine (DMA) are extremely small in fish meat immediately after harvesting but increase as freshness decreases (Taşkaya & Yaşar, 2018). Therefore, the TVB‐N value is accepted as a spoilage index for fish. The Food and Agricultural Organization (FAO) has indicated that sample with a TVB‐N value less than 25 mg N/100g is “perfect quality,” up to 30 mg N/100g is “good quality,” up to 35 mg N/100g is “marketable quality,” and greater than 35 mg N/100g is indicated as “spoiled” (FAO, 1986; Schormuller, 1968). It has also been demonstrated that fish meat with a TVB‐N content of 5–10 mg/100g is extremely fresh, whereas TVB‐N levels of 15–20 mg/100 g suggest early decay, and levels of 50 mg/100 g indicate a high degree of decay (Song et al., 2005). In the present study, the TVB‐N values of the FM and SSDM groups were 10 mg/% or less from days 0 to 4 during cold storage (Figure 2b). Upon storage at 4°C for 14 days, the values of TVB‐N increased gradually until 10 days of storage followed by a rapid increase from days 10 to 14 of storage. On day 10 of storage, the freshness of the SSDM6 group was the lowest at 15.05 mg/% when compared with the other SSDM groups. At 14 days, the TVB‐N values were the highest in the SSDM3 group (28.35 mg/%), whereas the lowest in the SSDM2 group (23.1 mg/%). The increase in TVB‐N is related to the formation of ammonia and trimethylamine induced by enzyme autolysis and bacterial spoilage. By contrast, the addition of sodium chloride inhibits enzyme autolysis in fish (Nooralabettu, 2008). In the present study, all the SSDM samples were within the limits during refrigerated storage for 14 days.

### 3.5 Fatty acid compositions

The compositions of fatty acids (FAs) of SSDM are summarized in Table 4. In general, marine fish contain higher PUFA content due to their diet, resulting in a high ratio of PUFA to SFA (P/S) (Osman, Suriah, & Law, 2001). In the present study, a broad range of FAs was detected in fresh and SSDM, with an abundance of palmitic acid (C16:0), heptadecanoic acid (17:0), palmitoleic acid (C16:1), oleic acid (C18:1n‐9), eicosapentaenoic acid (EPA, C20:5n‐3), and docosahexaenoic acid (DHA, C22:6n‐3). Ackman and Eaton (1966) demonstrated that palmitic acid was a major metabolite in fish. Moreover, the predominance of EPA and DHA, which prevent human coronary heart disease, is an adaptation to the low temperature of the marine environment, and thus, contribute to the maintenance of cell membrane fluidity (Farkas, 1979; Ruxton, Reed, Simpson, & Millington, 2004). In our study, it is worth mentioning that both fresh and SSDM contained a large proportion of SFA (48.17%–53.75% of total FAs) and PUFA (26.3%–42.91% of total FAs), especially EPA (17.72%–32.21% of total FAs), DHA (5.75%–11.15% of total FAs), and P/S (0.48–0.89). Cengiz, Ünlü, and Başhan (2010) reported SFA, MUFA, and PUFA levels of 48.94%, 41.34%, and 9.75% in Abu mullet (*Liza abu*) similar to our study. Köse, Koral, Özoğul, and Tufan (2010) also reported that the total values of SFA, MUFA, and PUFA in muscle samples of Pacific mullet were 29.59%, 29.26%, and 18.06%, respectively. Pollero et al. (1979) reported that the contents of DHA and EPA in a few marine fish and shellfish were related to the type of food, seasonal changes, and sexual cycle.

**Table 4:** Fatty acid compositions (g/100 g total fatty acids) of fresh and salted semidried mullet

	Groups									
	FM	SSDM1	SDM2	SSDM3	SSDM4	SSDM5	SSDM6	SSDM7	SSDM8	SSDM9
Fatty acid										
Butyric acid (C4:0)	—	—	—	—	—	—	—	—	—	—
Caproic acid (C6:0)	—	—	—	—	—	—	—	—	—	—
Caprylic acid (C8:0)	0.24 ± 0.01^d^	0.24 ± 0.01^d^	0.23 ± 0.03^d^	—	0.82 ± 0.02^a^	0.18 ± 0.01^e^	0.25 ± 0.01^d^	0.23 ± 0.02^d^	0.35 ± 0.02^b^	0.28 ± 0.01^c^
Capric acid (C10:0)	0.11 ± 0.02^c^	—	—	—	0.28 ± 0.01^a^	0.10 ± 0.01^c^	0.11 ± 0.03^c^	0.10 ± 0.02^c^	0.14 ± 0.01^b^	0.10 ± 0.01^c^
Undecanoic acid (C11:0)	—	—	—	—	—	—	—	—	—	—
Lauric acid (C12:0)	0.12 ± 0.01^c^	0.16 ± 0.03^a^	—	—	—	0.16 ± 0.04^a^	0.15 ± 0.02^ab^	0.15 ± 0.01^ab^	0.18 ± 0.03^a^	0.16 ± 0.01^a^
Tridecanoic acid (C13:0)	—	0.18 ± 0.03^a^	—	—	—	0.07 ± 0.01^c^	0.13 ± 0.02^b^	0.08 ± 0.01^c^	—	0.08 ± 0.02^c^
Myristic acid (C14:0)	7.35 ± 0.50^a^	6.41 ± 0.40^b^	5.88 ± 0.88^bc^	4.56 ± 0.14^d^	3.91 ± 0.31^e^	5.68 ± 0.16^c^	5.23 ± 0.03^c^	5.30 ± 0.05^c^	2.05 ± 0.03^f^	3.75 ± 0.25^e^
Pentadecanoic acid (C15:0)	0.81 ± 0.10^g^	1.47 ± 0.04^e^	2.14 ± 0.06^bc^	1.94 ± 0.04^d^	2.73 ± 0.05^a^	2.23 ± 0.03^b^	2.05 ± 0.11^c^	1.49 ± 0.05^e^	1.30 ± 0.05^f^	1.92 ± 0.02^d^
Palmitic acid (C16:0)	21.02 ± 1.14^b^	17.63 ± 1.08^c^	14.71 ± 0.30^e^	14.75 ± 0.52^e^	23.59 ± 0.42^a^	17.97 ± 0.22^c^	16.01 ± 0.21^d^	14.25 ± 0.24^e^	18.37 ± 0.15^c^	20.04 ± 0.92^b^
Heptadecanoic acid (C17:0)	9.09 ± 0.26^e^	15.15 ± 1.04^c^	23.74 ± 1.52^b^	21.66 ± 1.66^c^	6.59 ± 0.25^f^	12.04 ± 0.21^d^	15.13 ± 0.12^c^	26.63 ± 2.22^a^	12.74 ± 0.61^d^	11.35 ± 0.30^d^
Stearic acid (C18:0)	4.99 ± 0.72^cd^	4.54 ± 0.33^de^	2.30 ± 0.17^g^	3.93 ± 0.32^ef^	8.63 ± 0.63^a^	5.47 ± 0.20^c^	5.54 ± 0.17^c^	3.82 ± 0.11^f^	7.03 ± 0.20^b^	6.66 ± 0.14^b^
Arachidic acid (C20:0)	0.24 ± 0.01^e^	—	—	—	0.41 ± 0.02^c^	0.37 ± 0.03^d^	0.73 ± 0.05^a^	0.38 ± 0.02^cd^	0.68 ± 0.01^b^	0.39 ± 0.01^cd^
Heneicosanoic acid (C21:0)	—	—	—	—	—	—	—	—	—	—
Behenic acid (C22:0)	0.15 ± 0.01^e^	0.60 ± 0.02^b^	0.25 ± 0.02^d^	0.16 ± 0.01^e^	—	0.11 ± 0.01^ef^	0.48 ± 0.03^c^	0.66 ± 0.06^a^	0.09 ± 0.01^f^	0.12 ± 0.01^ef^
Tricosanoic acid (C23:0)	4.04 ± 0.24^e^	2.88 ± 0.11^fg^	3.28 ± 0.22^f^	3.83 ± 0.12^e^	6.78 ± 0.25^a^	4.59 ± 0.53^d^	3.82 ± 0.11^e^	2.52 ± 0.09^g^	5.34 ± 0.32^c^	5.98 ± 0.13^b^
Lignoceric acid (C24:0)	—	—	—	—	—	—	0.12 ± 0.01^a^	—	0.04 ± 0.01^c^	0.10 ± 0.01^b^
SFAs	48.17 ± 3.02^c^	49.25 ± 3.09^bc^	52.54 ± 3.20^abc^	50.82 ± 2.80^bc^	53.75 ± 1.96^ab^	48.98 ± 1.46^c^	49.77 ± 0.91^bc^	55.60 ± 2.90^a^	48.32 ± 1.45^c^	50.93 ± 1.84^bc^
Myristoleic acid (C14:1)	0.27 ± 0.03^a^	0.09 ± 0.01^d^	0.11 ± 0.01^cd^	—	—	0.13 ± 0.01^c^	0.19 ± 0.01^b^	0.12 ± 0.02^c^	—	—
cis−10‐Pentadecenoic acid (C15:1)	—	—	—	—	—	—	—	—	—	—
Palmitoleic acid (C16:1)	11.23 ± 0.52^ab^	11.63 ± 0.61^a^	10.46 ± 0.30^cd^	8.29 ± 0.15^d^	7.52 ± 0.11^e^	10.76 ± 0.13^bc^	8.80 ± 0.08^d^	11.37 ± 0.37^a^	3.69 ± 0.36^f^	11.18 ± 0.16^ab^
cis−10‐Heptadecenoic acid (C17:1)	—	0.10 ± 0.01^c^	0.30 ± 0.01^a^	0.17 ± 0.01^b^	—	0.07 ± 0.01^d^	0.11 ± 0.02^c^	0.28 ± 0.03^a^	0.05 ± 0.01^d^	—
Elaidic acid (C18:1n9t)	0.21 ± 0.01^a^	0.11 ± 0.01^d^	—	—	—	0.20 ± 0.01^a^	0.18 ± 0.01^b^	0.09 ± 0.01^e^	0.13 ± 0.01^c^	—
Oleic acid (C18:1n9c)	11.37 ± 1.12^a^	7.71 ± 0.21^b^	5.25 ± 0.38^ef^	5.10 ± 0.08^ef^	6.02 ± 0.19^cd^	7.51 ± 0.21^b^	6.53 ± 0.40^c^	5.74 ± 0.13^de^	3.81 ± 0.10^g^	4.87 ± 0.21^f^
cis−11‐Eicosenoic acid (C20:1)	—	0.23 ± 0.02^d^	—	—	0.12 ± 0.01^f^	0.58 ± 0.03^b^	0.43 ± 0.01^c^	0.16 ± 0.01^e^	0.17 ± 0.02^e^	0.63 ± 0.03^a^
Erucic acid (C22:1n9)	—	0.23 ± 0.03^f^	0.35 ± 0.01^cd^	0.48 ± 0.01^b^	0.17 ± 0.01^h^	0.28 ± 0.01^e^	0.37 ± 0.03^c^	0.33 ± 0.02^d^	0.77 ± 0.01^a^	0.20 ± 0.01^g^
Nervonic acid (C24:1)	—	—	—	—	—	0.13 ± 0.01^b^	0.17 ± 0.01^a^	—	0.13 ± 0.01^b^	0.18 ± 0.01^a^
MUFAs	23.08 ± 1.68^a^	20.10 ± 0.90^b^	16.47 ± 0.71^d^	14.05 ± 0.25^e^	13.83 ± 0.32^e^	19.67 ± 0.42^b^	16.78 ± 0.57^cd^	18.10 ± 0.59^c^	8.76 ± 0.52^f^	17.05 ± 0.42^cd^
Linolelaidic acid (C18:2n6t)	—	—	—	—	—	—	—	—	—	—
Linoleic acid (C18:2n6c)	0.87 ± 0.03^c^	1.09 ± 0.02^b^	0.47 ± 0.01^f^	0.53 ± 0.03^ef^	0.76 ± 0.01^cd^	1.22 ± 0.20^ab^	0.91 ± 0.23^c^	1.29 ± 0.02^a^	0.66 ± 0.05^de^	1.22 ± 0.01^ab^
cis−11,14‐Eicosadienoic acid C20:2)	—	—	—	—	0.16 ± 0.01^c^	0.21 ± 0.01^b^	0.28 ± 0.01^a^	0.13 ± 0.01^d^	0.16 ± 0.01^c^	0.20 ± 0.01^b^
cis−13,16‐Docosadienoic acid (C22:2)	—	—	—	—	—	0.03 ± 0.01^c^	0.05 ± 0.01^b^	0.06 ± 0.01^a^	—	—
ɣ‐Linolenic acid (C18:3n6)	0.18 ± 0.01^e^	0.49 ± 0.06^b^	0.34 ± 0.02^d^	0.41 ± 0.01^c^	0.57 ± 0.06^a^	0.51 ± 0.03^b^	0.39 ± 0.02^cd^	0.40 ± 0.01^c^	0.21 ± 0.04^e^	0.61 ± 0.01^a^
Linolenic acid (C18:3n3)	0.33 ± 0.02^e^	0.82 ± 0.02^d^	0.90 ± 0.07^d^	0.80 ± 0.05^d^	0.31 ± 0.01^e^	1.04 ± 0.12^c^	1.33 ± 0.13^a^	1.15 ± 0.13^bc^	1.25 ± 0.08^ab^	0.88 ± 0.04^d^
cis−8, 11, 14‐Eicosatrienoic acid (C20:3n6)	0.19 ± 0.01^cd^	0.20 ± 0.01^c^	0.15 ± 0.02^e^	0.19 ± 0.01^cd^	0.29 ± 0.03^a^	0.28 ± 0.01^a^	0.24 ± 0.02^b^	0.16 ± 0.01^de^	0.27 ± 0.03^ab^	0.27 ± 0.01^ab^
cis−11,14,17‐Eicosatrienoic acid (C20:3n3)	—	—	—	—	—	—	—	—	—	—
Arachidonic acid (C20:4n6)	—	—	—	—	—	—	—	—	—	—
cis−5,8,11,14,17‐Eicosapentaenoic acid (C20:5n3)	20.49 ± 0.20^e^	20.94 ± 0.23^d^	23.36 ± 0.06^cd^	27.05 ± 0.21^b^	19.17 ± 0.11^f^	17.72 ± 0.21^h^	20.72 ± 0.03^de^	23.12 ± 0.11^c^	32.21 ± 0.10^a^	18.36 ± 0.32^g^
cis−4,7,10,13,16,19‐Docosahexaenoic acid (C22:6n3)	6.70 ± 0.02^f^	6.87 ± 0.56^e^	5.77 ± 0.14^g^	6.14 ± 0.03^g^	11.15 ± 0.07^a^	10.35 ± 0.04^b^	9.53 ± 0.12^c^	5.75 ± 0.01^g^	8.15 ± 0.32^d^	10.49 ± 0.31^b^
PUFAs	28.76 ± 0.29^f^	30.65 ± 0.91^edf^	30.99 ± 0.322^de^	35.14 ± 0.34^b^	32.43 ± 0.30^cd^	31.35 ± 0.63^cde^	33.45 ± 0.57^bc^	26.30 ± 3.34^ef^	42.91 ± 0.63^a^	32.03 ± 0.71^cde^

Note

—: represents that the corresponding amino acid was not detected.

Values represent mean ± standard deviation (*SD*) (*n* = 5). Different superscript letters within each row represent significant differences (*p* < .05).

Abbreviations: FM, fresh mullet; MUFAs, monounsaturated fatty acids; PUFAs, polyunsaturated fatty acids; SFAs, saturated fatty acids;

SSDM, salted semidried mullet.

### 3.6 Amino acid compositions

The constituent amino acids of fresh and SSDM are shown in Table 5. The total content of amino acids in the FM group was 16,841 mg/100 g. On the other hand, the total amino acid content in SSDM 4 group was the highest at 28,894 mg/100 g and the lowest at 13,943 mg/100 g in SSDM 3 group. Both FM and SSDM groups contained a large proportion of essential amino acids (EAA) such as lysine and leucine, and a few nonessential amino acids (NEAA) including glutamic acid, aspartic acid, alanine, and arginine. In addition, the content of each constituent amino acid was increased in the SSDM groups except SSDM1 and SSDM3 compared with the FM group. In the present study, the total content of free amino acids in the FM group was 315 mg/100 g. On the other hand, the total content of free amino acids in the SSDM2 (326 mg/100 g), SSDM3 (334 mg/100 g), and SSDM5 (415 mg/100 g) groups was higher than in the FM group (Table 6). Among the free amino acids, taurine, glycine, and alanine were the most abundant in fresh and SSDM samples. Joo (2011) reported decreased levels of a few amino acids such as taurine, serine, and glycine, and an increase in alanine, glutamate, valine, threonine, leucine, and lysine content of salted and dried products from brown croaker during storage at 5°C for 28 days.

**Table 5:** Constituent amino acids (mg/100g) of fresh and salted semidried mullet

	Groups									
	FM	SSDM1	SSDM2	SSDM3	SSDM4	SSDM5	SSDM6	SSDM7	SSDM8	SSDM9
Component										
Aspartic acid	1,813.678 ± 24.13^g^	1,775.254 ± 17.1^h^	2,306.168 ± 32.35^c^	2,049.727 ± 25.39^f^	2,159.587 ± 27.77^e^	2,023.731 ± 23.42^f^	2,237.252 ± 23.22^d^	2,064.277 ± 49.29^f^	3,001.398 ± 28.28^a^	2,766.041 ± 14.29^b^
Threonine	827.093 ± 26.56^g^	803.931 ± 12.84^g^	1,043.38 ± 11.62^d^	653.218 ± 11.39^h^	1,466.321 ± 28.47^a^	894.132 ± 61.94^f^	984.802 ± 57.58^de^	929.602 ± 39.41^ef^	1,361.143 ± 24.15^b^	1,271.695 ± 47.47^c^
Serine	760.735 ± 40.89^g^	742.31 ± 30.95^g^	983.876 ± 28.35^d^	616.064 ± 29.03^h^	1,371.621 ± 53.36^a^	850.587 ± 11.07^f^	930.234 ± 21.74^de^	875.642 ± 43.38^ef^	1,269.03 ± 31.81^b^	1,181.086 ± 51.54^c^
Glutamic acid	2,572.491 ± 28.61^h^	2,514.203 ± 10.99^h^	3,310.782 ± 11.12^d^	2,105.261 ± 26.71^i^	4,608.049 ± 54.15^a^	2,872.085 ± 33.56^g^	3,151.853 ± 16.99^e^	2,982.226 ± 17.67^f^	4,293.84 ± 94.32^b^	3,875.019 ± 58.17^c^
Proline	660.372 ± 30.12^ef^	608.47 ± 34.92^f^	816.056 ± 15.79^c^	491.68 ± 72.46^g^	1,094.788 ± 23.53^a^	693.09 ± 30.04^de^	743.342 ± 41.75^d^	717.444 ± 35.17^de^	1,078.831 ± 26.68^a^	962.945 ± 25.73^b^
Glycine	882.284 ± 12.03^g^	853.766 ± 14.55^g^	1,317.211 ± 16.96^d^	779.149 ± 19.93^h^	1,818.068 ± 28.97^a^	1,128.265 ± 27.6^e^	1,098.905 ± 69.36^e^	1,012.573 ± 14.32^f^	1,645.502 ± 42.92^b^	1,486.003 ± 29.44^c^
Alanine	1,083.535 ± 82.99^g^	1,039.528 ± 24.16^g^	1,429.729 ± 12.36^d^	870.641 ± 16.42^h^	1,951.294 ± 13.63^a^	1,228.81 ± 39.28^f^	1,327.913 ± 27.4^b^	1,216.656 ± 16.47^f^	1,839.099 ± 36.42^b^	1,643.217 ± 29.37^c^
Valine	871.48 ± 39.93^f^	840.587 ± 21.01^f^	1,064.173 ± 27.02^d^	661.134 ± 23.94^g^	1,533.76 ± 31.79^a^	953.657 ± 15.88^e^	1,034.383 ± 20.54^d^	970.725 ± 50.13^e^	1,398.838 ± 31.98^b^	1,269.858 ± 50.03^c^
Methionine	532.438 ± 10.58^e^	519.558 ± 19.34^e^	679.515 ± 41.36^c^	428.043 ± 24.09^f^	972.173 ± 21.97^a^	624.252 ± 19.8^d^	648.803 ± 31.58^cd^	656.575 ± 23.24^cd^	884.956 ± 29.79^b^	865.067 ± 21.71^b^
Isoleucine	773.022 ± 34.17^f^	759.291 ± 15.47^f^	936.714 ± 36.46^d^	588.029 ± 16.81^g^	1,382.703 ± 13.01^a^	844.906 ± 15.15^e^	927.431 ± 15.07^d^	851.872 ± 28.48^e^	1,252.373 ± 12.83^b^	1,149.967 ± 12.71^c^
Leucine	1,415.019 ± 14.16^g^	1,381.396 ± 14.18^g^	1,747.677 ± 23.71^d^	1,117.746 ± 28.23^h^	2,515.052 ± 26.94^a^	1,563.914 ± 33.98^f^	1,720.668 ± 20.41^d^	1,606.029 ± 16.97^e^	2,322.174 ± 21.21^b^	2,140.605 ± 14.32^c^
Tyrosine	622.04 ± 21.19^h^	604.275 ± 12.51^h^	785.543 ± 15.21^d^	489.202 ± 13.94^i^	1,115.995 ± 17.12^a^	683.081 ± 19.99^g^	746.638 ± 11.09^e^	711.771 ± 13.55^f^	1,015.085 ± 14.1^b^	974.414 ± 22.25^c^
Phenylalanine	714.28 ± 13.99^f^	698.004 ± 28.72^f^	918.299 ± 21.08^d^	565.396 ± 24.34^g^	1,309.085 ± 20.82^a^	793.063 ± 14.86^e^	889.026 ± 25.18^d^	785.933 ± 24.67^e^	1,220.619 ± 12.24^b^	1,141.297 ± 22.58^c^
Histidine	625.087 ± 23.23^de^	600.426 ± 27.11^e^	649.005 ± 16.94^cd^	406.037 ± 14.88^f^	939.664 ± 22.03^a^	588.255 ± 14.4^e^	668.319 ± 26.42^c^	589.593 ± 18.07^e^	862.726 ± 12.62^b^	838.451 ± 27.26^b^
Lysine	1,657.833 ± 26.01^f^	1,629.598 ± 14.08^f^	1,980.737 ± 10.48^d^	1,308.321 ± 19.51^g^	2,790.476 ± 14.26^a^	1,852.83 ± 18.87^e^	1,984.673 ± 17.96^d^	1,833.861 ± 31.6^e^	2,568.678 ± 8.62^b^	2,255.515 ± 7.34^c^
Arginine	1,029.655 ± 28.37^h^	996.217 ± 9.09^i^	1,325.124 ± 11.03^d^	813.974 ± 13.75^j^	1,866.038 ± 17^a^	1,131.61 ± 11.78^g^	1,257.621 ± 11.34^e^	1,165.568 ± 28.31^f^	1,712.567 ± 11.97^b^	1,595.311 ± 20.1^c^
Total	16,841.044 ± 457.01^g^	16,366.813 ± 307.08^g^	21,293.991 ± 331.92^d^	13,943.623 ± 380.9^h^	28,894.674 ± 414.89^a^	18,726.267 ± 391.67^f^	20,351.864 ± 437.69^e^	18,970.348 ± 450.8^f^	27,726.857 ± 440.02^b^	25,416.491 ± 454.37^c^

Note

—: represents that the corresponding amino acid was not detected.

Values represent mean ± standard deviation (*SD*) (*n* = 5). Different superscript letters within each row represent significant differences (*p* < .05).

Abbreviations: FM, fresh mullet); SSDM, salted semidried mullet.

**Table 6:** Free amino acids (mg/100g) of fresh and salted semidried mullet

	Groups									
	FM	SSDM1	SDM2	SSDM3	SSDM4	SSDM5	SSDM6	SSDM7	SSDM8	SDM9
Component										
Phosphoserine	—	—	—	—	—	—	—	—	—	—
Taurine	126.555 ± 7.3^a^	75.798 ± 3.93^de^	70.774 ± 1.98^f^	89.239 ± 5.75^c^	61.501 ± 1.26^g^	78.549 ± 1.39^d^	112.154 ± 1.85^b^	71.306 ± 1.79^f^	81.642 ± 1.48^d^	38.224 ± 3.96^h^
Phosphoethanolamine	—	—	—	—	—	—	—	—	—	—
Urea	—	—	—	—	—	—	—	—	—	—
Aspartic acid	—	—	—	—	—	—	—	—	—	—
Hydroxyproline	—	—	—	—	—	—	—	—	—	—
Threonine	5.400 ± 0.4^ef^	7.897 ± 1.02^cd^	8.959 ± 1.74^bc^	9.141 ± 1.12^bc^	6.332 ± 0.27^de^	10.204 ± 0.35^ab^	5.508 ± 0.29^ef^	9.515 ± 0.49^bc^	11.604 ± 1.75^a^	4.239 ± 0.28^f^
Serine	3.216 ± 0.2^c^	3.341 ± 0.18^c^	6.338 ± 0.31^a^	6.542 ± 0.21^a^	3.338 ± 0.35^c^	6.742 ± 0.52^ab^	5.343 ± 0.34^b^	5.279 ± 0.4^b^	5.087 ± 0.29^b^	1.855 ± 0.82^d^
Asparagine	—	—	—	—	—	—	—	—	—	—
Glutamic acid	2.202 ± 0.19^c^	2.031 ± 0.15^c^	3.401 ± 0.38^a^	3.495 ± 0.38^a^	1.539 ± 0.21^d^	2.845 ± 0.35^b^	1.830 ± 0.14^cd^	2.016 ± 0.02^c^	3.477 ± 0.25^a^	2.159 ± 0.15^c^
Sarcosine	—	—	—	—	—	—	—	—	—	—
α‐Aminoadipic acid	—	—	—	—	—	—	—	—	—	—
Proline	3.011 ± 0.22^e^	12.969 ± 2.7^b^	12.874 ± 1.34^b^	8.354 ± 0.29^cd^	8.356 ± 0.7^cd^	16.548 ± 1.42^a^	7.969 ± 0.9^cd^	14.352 ± 2.28^ab^	10.473 ± 0.9^c^	6.593 ± 0.56^d^
Glycine	85.509 ± 2.48^c^	23.172 ± 1.95^g^	90.735 ± 1.74^b^	83.722 ± 1.55^c^	79.518 ± 1.46^d^	109.172 ± 1.51^a^	41.141 ± 0.54^e^	24.153 ± 1.16^g^	42.100 ± 2.07^e^	31.914 ± 3.69^f^
Alanine	35.417 ± 3.08^d^	28.377 ± 1.11^e^	53.202 ± 1.93^b^	51.285 ± 1.02^b^	28.371 ± 1.48^e^	74.348 ± 1.36^a^	40.984 ± 1.56^c^	38.391 ± 1.22^cd^	50.437 ± 2.42^b^	13.741 ± 2.47^f^
Citrulline	—	—	—	—	—	—	—	—	—	—
α—Aminobutyric acid	—	—	—	—	—	—	—	—	—	—
Valine	5.424 ± 1.16^de^	10.668 ± 1.75^c^	13.846 ± 1.5^abc^	12.313 ± 2.05^bc^	7.037 ± 1.14^d^	15.249 ± 1.47^ab^	11.689 ± 2.42^c^	15.716 ± 2.16^a^	16.824 ± 2.59^a^	2.615 ± 0.57^e^
Cystine	—	—	—	—	—	—	—	—	—	—
Methionine	2.234 ± 0.21^e^	6.018 ± 1.05^bc^	3.962 ± 0.95^d^	4.620 ± 0.68^cd^	2.486 ± 0.29^e^	6.537 ± 1.31^b^	5.591 ± 0.6^bc^	6.364 ± 0.9^b^	8.991 ± 0.92^a^	1.126 ± 0.08^e^
Isoleucine	3.851 ± 0.76^c^	5.928 ± 1.9^b^	7.294 ± 0.89^b^	9.180 ± 1.18^a^	2.486 ± 0.46^cd^	6.537 ± 0.65^b^	5.591 ± 0.98^b^	6.364 ± 0.35^b^	8.991 ± 0.88^a^	1.126 ± 0.13^d^
Leucine	5.635 ± 0.41^d^	13.845 ± 1.85^b^	11.447 ± 0.45^c^	15.903 ± 1.56^a^	—	—	—	—	—	—
Tyrosine	3.493 ± 0.4^d^	6.318 ± 0.4^b^	4.711 ± 0.67^cd^	6.629 ± 0.9^b^	4.602 ± 0.48^cd^	10.188 ± 1.25^a^	5.864 ± 0.75^bc^	8.967 ± 1.27^a^	9.647 ± 0.61^a^	1.921 ± 0.21^e^
Phenylalanine	1.598 ± 0.4^cd^	7.778 ± 0.88^f^	6.636 ± 0.61^d^	4.308 ± 0.56^b^	7.935 ± 0.6^e^	20.714 ± 0.9^a^	12.722 ± 1.68^d^	19.166 ± 1.48^bc^	20.448 ± 0.44^cd^	3.724 ± 0.48^f^
β‐Alanine	5.051 ± 0.17^cd^	1.556 ± 0.51^f^	4.799 ± 0.46^s^	6.310 ± 0.32^b^	2.395 ± 0.37^e^	7.538 ± 0.4^a^	4.865 ± 0.02^d^	5.794 ± 0.78^bc^	5.342 ± 0.56^cd^	1.098 ± 0.28^f^
β‐Aminoisobutyric acid	0.826 ± 0.07^f^	0.741 ± 0.04^f^	0.725 ± 0.09^f^	—	4.132 ± 0.28^d^	7.528 ± 0.48^b^	5.392 ± 0.58^c^	9.565 ± 0.56^a^	5.387 ± 0.67^c^	2.752 ± 0.42^e^
γ‐Amino‐n‐butyric acid	1.577 ± 0.31^e^	0.464 ± 0.23^f^	0.360 ± 0.16^f^	0.453 ± 0.12^f^	4.589 ± 0.03^c^	6.781 ± 0.67^a^	5.491 ± 0.77^b^	2.519 ± 0.29^d^	4.935 ± 0.41^bc^	2.989 ± 0.27^d^
Histidine	18.675 ± 1.46^b^	39.167 ± 1.16^a^	16.922 ± 0.23^c^	15.751 ± 0.74^d^	0.593 ± 0.15^e^	1.217 ± 0.2^e^	0.841 ± 0.07^e^	0.573 ± 0.23^e^	1.042 ± 0.12^e^	—
1‐Methylhistidine	—	—	—	—	0.240 ± 0.01^cd^	0.705 ± 0.11^bc^	0.345 ± 0.05^cd^	3.583 ± 0.79^a^	0.918 ± 0.21^b^	0.297 ± 0.08^cd^
3‐Methylhistidine	—	—	—	—	16.798 ± 1.8^d^	30.968 ± 2.75^a^	32.546 ± 1.46^a^	26.777 ± 2.67^b^	1.665 ± 0.11^e^	19.805 ± 0.77^c^
Carnosine	—	—	—	—	—	—	—	—	—	—
Anserine	—	—	—	—	—	—	—	—	—	—
Tryptophan	—	—	—	—	—	—	—	—	—	—
Hydroxylysine	—	—	—	—	—	—	—	—	—	—
Ornithine	0.552 ± 0.13^c^	0.995 ± 0.2^b^	1.391 ± 0.25^a^	0.484 ± 0.16^c^	—	—	—	—	—	—
Lysine	4.061 ± 0.21^b^	4.507 ± 0.27^a^	4.181 ± 0.17^b^	4.205 ± 0.3^b^	—	—	—	—	—	—
Arginine	1.242 ± 0.15^e^	6.811 ± 0.14^a^	3.627 ± 0.17^b^	2.895 ± 0.27^c^	2.353 ± 0.26^cd^	6.759 ± 0.54^a^	2.753 ± 0.42^cd^	2.215 ± 0.23^d^	1.077 ± 0.26^e^	1.354 ± 0.34^e^
Total	315.529 ± 24.73^bc^	258.382 ± 32.42^d^	326.184 ± 23.56^bc^	334.829 ± 25.77^b^	246.689 ± 15.86^d^	415.716 ± 20.53^a^	312.636 ± 21.79^bc^	270.007 ± 18.54^d^	285.315 ± 13.08^cd^	140.629 ± 19.76^e^

Note

—: represents that the corresponding amino acid was not detected.

Values represent mean ± standard deviation (*SD*) (*n* = 5). Different superscript letters within each row represent significant differences (*p* < .05).

Abbreviations: FM, fresh mullet; SSDM, salted semidried mullet.

## 4 SDS‐PAGE

**Figure 3:**
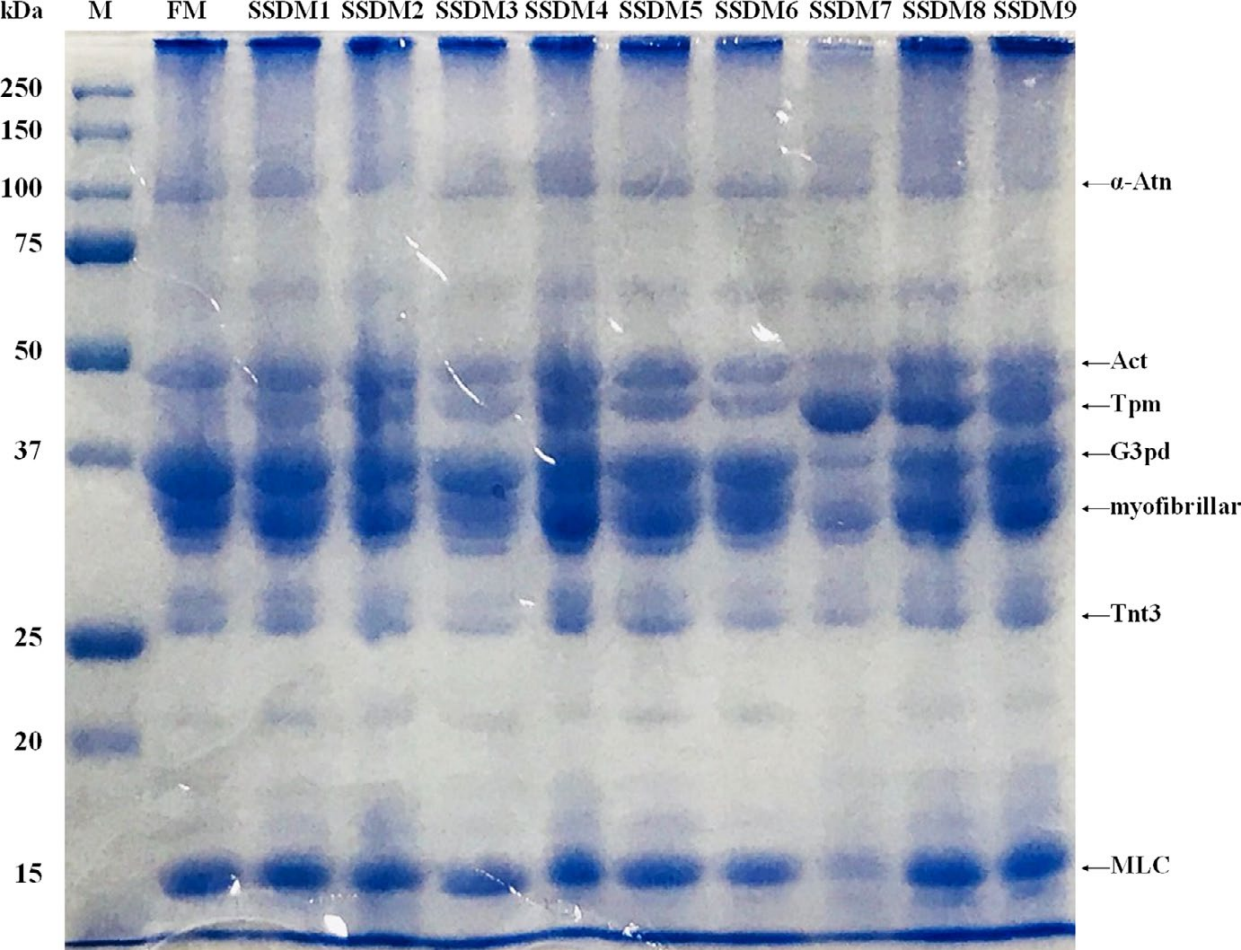
SDS‐PAGE pattern of the fresh mullet (FM) and salted semidried mullet (SSDM) samples M, broad range protein marker; kDa, molecular weight of proteins. Act, actin; G3pd, glyceraldehyde‐3‐phosphate dehydrogenase fragment; MLC, light chain of myosin; Tnt3, troponin T type 3b protein fragment; Tpm, tropomyosin; α‐Atn, α‐actinin

The muscle fiber protein of fish meat generally constitutes 60%–70% of the muscle protein. It contributes to the physical properties of dietary protein as well as playing a role in muscle tissue formation as a structural protein. It has been known that the reactivity of the proteolytic enzyme to the myofibrillar protein depends on the freshness and quality of fish (Seki & Watanabe, 1984). The electrophoretic profiles of SSDM samples are shown in Figure 3. No remarkable changes in protein profiles were observed in fresh and SSDM. The major protein bands observed in fresh mullets and SSDM included α‐actinin (α‐Atn), actin (Act), tropomyosin (Tpm), glyceraldehyde‐3‐phosphate dehydrogenase fragment (G3pd), myofibrils, troponin T type 3b protein fragment (Tnt3), and light chain of myosin (MLC) (Figure 3). In particular, a new protein identified as Tpm was detected in all SSDM groups, although the band intensities of Act, Tpm, myofibrillar, and Tnt3 proteins in SSDM3, SSDM6, and SSDM7 groups were slightly decreased compared with the FM group. Similarly, Joo (2011) reported that the electrophoretic pattern of salted and dried brown croaker products was altered slightly by different salting conditions and storage periods. These results may be attributed to conformational changes of proteins and increased intracellular enzymes released by different salting and processing methods.

### 4.1 Total coliform and *Escherichia coli* levels

In general, microbial contamination of foods may occur due to mishandling during distribution, processing, and storage (Hashem & Alamri, 2010). Table 7 summarizes the initial values of the total coliform and *E. coli* in fresh and SSDM samples prepared using different salt concentrations, drying and pretreatment methods. In the present study, the initial level of total coliforms in all samples ranged from 1.00 to 2.82 log cfu/g, respectively. In several processed fish products, these microbial groups have been already reported and traced to the raw materials or contamination during processing (Hsu et al., 2009; Kung et al., 2008). On the other hand, none of the fresh and SSDM samples contained *E. coli*. It was possible that *E. coli* levels were suppressed by salting process. These results are in agreement with previous studies of high levels of aerobic plate count and total coliforms in dried milkfish produced by sun drying and containing low salt (1.2%–2.3% of NaCl) (Hsu et al., 2009). However, aerobic plate counts, *E. coli*, and total coliforms were not detectable in dried milkfish produced by elevated salts (>2.5%) (Hwang et al., 2012).

**Table 7:** Total coliforms and *E. Coli* contents (log cfu/g) in salted semidried mullet

Groups	Coliforms	*E. Coli*
FM	2.11 ± 0.01^e^	ND
SSDM1	2.18 ± 0.02^c^	ND
SSDM2	1.78 ± 0.01^g^	ND
SSDM3	1.00 ± 0.01^j^	ND
SSDM4	2.82 ± 0.02^a^	ND
SSDM5	2.26 ± 0.03^b^	ND
SSDM6	1.48 ± 0.01^i^	ND
SSDM7	2.15 ± 0.01^d^	ND
SSDM8	2.00 ± 0.01^f^	ND
SSDM9	1.48 ± 0.01^h^	ND

Note

Values represent mean ± standard deviation (*SD*) (*n* = 5). Different superscript letters within each column represent significant differences (*p* < .05).

Abbreviations: FM, fresh mullet; ND, not detectable (level less than 1 log cfu/g); SSDM, salted semidried mullet.

### 4.2 Changes in total bacteria per storage period

Table 8 shows the changes in total microbial counts of SSDM during storage at 4°C for 14 days. In general, seafood is prone to rapid spoilage by microorganisms after harvest due to high moisture content. The components of seafood are degraded by several biochemical reactions, which lead to a shorter shelf life and loss of quality (Akonor et al., 2016). When the total bacterial count reaches about 5 to 6 log CFU/g, it is considered as an early stage of spoilage, and levels of 7 log CFU/g render food unfit for consumption (Lee, Moon, & Park, 2000). International Commission for Microbiological Standards of Foods (ICMSF, 1988) also recommends that raw fish and their products carrying a total microbial count in excess of 10^7^ should be considered as unacceptable. In this study, the initial total microbial counts of fresh and SSDM samples ranged from 3.41 to 5.30 log cfu/g, which was within acceptable limits. In the present study, the FM group showed the lowest total microbial count on day 0, but the total microbial counts were increased rapidly than in the other SSDM groups within the storage period. The total microbial counts in the FM group markedly increased up to 8.88 log cfu/g (increasing rate: 5.4 log cfu/g) after 14 days of refrigerated storage compared to levels of 7.76 log cfu/g (increasing rate: 2.6 log cfu/g) in SSDM4, 8.41 log cfu/g (increasing rate: 3.1 log cfu/g) in SSDM9, 7.38 log cfu/g (increasing rate: 3.4 log cfu/g) in SSDM5, and 7.69 log cfu/g (increasing rate: log cfu/g) in SSDM1, respectively. Similar to our results, Siriskar et al. (2013) reported that the salted and pressed anchovies showed an initial microbial count of 2 × 10^2^ log CFU/g, which increased up to 6.4 × 10^3^ log CFU/g during 5 weeks of storage. In our study, the rapid increase in total microbial counts during storage of FM group may be attributed to the high moisture content, *Aw*, and low salinity compared with those of the SSDM groups.

**Table 8:** Changes in total microbial counts (log cfu/g) of salted semidried mullet during storage at 4°C for 14 days

Groups	Storage period (days)							
	0	2	4	6	8	10	12	14
FM	3.41 ± 0.01^i^	5.38 ± 0.01^b^	6.91 ± 0.01^b^	8.49 ± 0.02^a^	8.76 ± 0.03^a^	9.00 ± 0.03^a^	9.34 ± 0.02^a^	8.88 ± 0.01^b^
SSDM1	4.23 ± 0.01^d^	4.85 ± 0.02^e^	7.38 ± 0.01^a^	7.23 ± 0.01^c^	7.48 ± 0.03^f^	7.51 ± 0.07^h^	7.56 ± 0.01^h^	7.69 ± 0.02^h^
SSDM2	3.92 ± 0.01^g^	4.94 ± 0.02^d^	6.20 ± 0.01^e^	6.91 ± 0.01^e^	7.71 ± 0.02^e^	8.26 ± 0.05^d^	8.32 ± 0.02^d^	8.36 ± 0.01^e^
SSDM3	4.56 ± 0.02^c^	4.32 ± 0.01^g^	6.71 ± 0.01^d^	6.83 ± 0.02^f^	7.79 ± 0.01^d^	8.00 ± 0.01^ef^	8.18 ± 0.01^e^	8.11 ± 0.01^f^
SSDM4	5.12 ± 0.02^b^	4.81 ± 0.01^f^	6.11 ± 0.01^f^	6.73 ± 0.03^g^	7.51 ± 0.01^f^	7.59 ± 0.05^g^	7.91 ± 0.01^g^	7.76 ± 0.02^g^
SSDM5	3.91 ± 0.01^g^	3.08 ± 0.02^j^	4.43 ± 0.01^j^	6.30 ± 0.01^h^	6.59 ± 0.02^g^	6.97 ± 0.01^i^	7.08 ± 0.03^i^	7.38 ± 0.02^i^
SSDM6	4.20 ± 0.01^e^	3.11 ± 0.01^i^	4.93 ± 0.01^h^	7.15 ± 0.06^d^	7.89 ± 0.08^c^	8.41 ± 0.04^b^	8.61 ± 0.01^c^	8.75 ± 0.05^c^
SSDM7	3.76 ± 0.01^h^	3.20 ± 0.01^h^	5.79 ± 0.01^g^	6.72 ± 0.02^g^	7.51 ± 0.01^f^	8.04 ± 0.02^e^	8.07 ± 0.03^f^	8.08 ± 0.05^f^
SSDM8	4.00 ± 0.01^f^	4.99 ± 0.01^c^	4.67 ± 0.02^i^	6.08 ± 0.04^i^	7.78 ± 0.02^d^	8.34 ± 0.03^c^	8.82 ± 0.01^b^	9.08 ± 0.03^a^
SSDM9	5.30 ± 0.01^a^	5.51 ± 0.01^a^	6.89 ± 0.01^c^	7.38 ± 0.01^b^	8.02 ± 0.01^b^	7.96 ± 0.01^f^	8.30 ± 0.06^d^	8.41 ± 0.03^d^

Note

Values represent mean ± standard deviation (*SD*) (*n* = 5). Different superscript letters within each column represent significant differences (*p* < .05).

Abbreviations: FM, fresh mullet; SSDM, salted semidried mullet.

## 5 CONCLUSIONS

This study represents the first report of physicochemical, nutritional, and sanitary properties of SSDM produced with different pretreatment methods including salting and drying at refrigerated temperatures. The different pretreatment techniques affected the TVB‐N content and lipid oxidation parameters such as POV, AV, and TBA and TBARS. Compared with the increasing microbial levels in FM group during storage, the SSDM groups showed a decrease in microbial content. Our findings suggested that the pretreatment method was one of the important factors in determining the physicochemical and nutritional properties, and the hygienic quality of SSDM products during refrigerated storage. In the present study, we confirmed that the SSDM produced by traditional methods improved the storage period significantly, unlike the fresh mullet. However, there is a need to simplify and standardize the traditional manufacturing methods and conditions to produce efficient salted semidried fish products.

## CONFLICT OF INTEREST

The authors declare that they do not have any conflict of interest.

## ETHICAL STATEMENT

This study does not involve any human testing.

## References

[fsn31270-bib-0001] Ackman, R. G. , & Eaton, C. A. (1966). Some commercial Atlantic herring oils; fatty acid composition. Journal of the Fisheries Research Board of Canada, 23(7), 991–1006. 10.1139/f66-092

[fsn31270-bib-0002] Akbary, P. (2019). Growth yield, carcass traits, biochemical and non‐ specific immune parameters in grey mullet, *Mugil cephalus* Linnaeus, 1758 under cyclic starvation and L‐ carnitine supplementation. Iranian Journal of Fisheries Sciences, 18(1), 15–29. 10.22092/ijfs.2018.117512

[fsn31270-bib-0003] Akonor, P. T. , Ofori, H. , Dziedzoave, N. T. , & Kortei, N. K. (2016). Drying characteristics and physical and nutritional properties of shrimp meat as affected by different traditional drying techniques. International Journal of Food Science, 2016, 1–5. 10.1155/2016/7879097 PMC4808551 27034924

[fsn31270-bib-0004] Ali, M. , Imran, M. , Nadeem, M. , Khan, M. K. , Sohaib, M. , Suleria, H. A. R. , & Bashir, R. (2019). Oxidative stability and Sensoric acceptability of functional fish meat product supplemented with plant‐based polyphenolic optimal extracts. Lipids in Health and Disease, 18(1), 35 10.1186/s12944-019-0982-y 30704486 PMC6357494

[fsn31270-bib-0005] Amerine, M. A. , Panborn, R. M. , & Roessler, E. B. (1965). Principals of sensory evaluation of food (pp. 338–339). London, NY: Academic Press.

[fsn31270-bib-0006] AOAC (2012). Official methods of analysis, 19th ed Arlington, VA: Association of Official Analytical Chemists.

[fsn31270-bib-0007] Bligh, E. G. , & Dyer, W. J. (1959). A rapid method of lipid extraction and purification. Canadian Journal of Biochemistry and Physiology, 37(8), 911–917. 10.1139/o59-099 13671378

[fsn31270-bib-0008] Bouic, P. J. (2001). The role of phytosterols and phytosterolins in immune modulation: A review of the past 10 years. Current Opinion in Clinical Nutrition & Metabolic Care, 4(6), 471–475. 10.1097/00075197-200111000-00001 11706278

[fsn31270-bib-0009] Cai, L. , Wu, X. , Dong, Z. , Li, X. , Yi, S. , & Li, J. (2014). Physicochemical responses and quality changes of red sea bream (Pagrosomus major) to gum arabic coating enriched with ergothioneine treatment during refrigerated storage. Food Chemistry, 160, 82–89. 10.1016/j.foodchem.2014.03.093 24799212

[fsn31270-bib-0010] Çelik, U. , Altielataman, C. , Dincer, T. , & Acarli, D. (2012). Comparison of fresh and dried flathead grey mullet (*Mugil cephalus*, Linnaeus 1758) caviar by means of proximate composition and quality changes during refrigerated storage at 4±2°C. Turkish Journal of Fisheries and Aquatic Sciences, 12, 1–5. 10.4194/1303-2712-v12_1_01

[fsn31270-bib-0011] Cengiz, E. İ. , Ünlü, E. , & Başhan, M. (2010). Fatty acid composition of total lipids in muscle tissues of nine. Turkish Journal of Biology, 34, 433–438. 10.3906/biy-0903-19

[fsn31270-bib-0012] Cho, S. J. , Rhee, C. O. , & Kim, D. Y. (1989). Study on the processing and compositions of salted and dried mullet roe. Korean Journal of Food Science and Technology, 21, 242–251.

[fsn31270-bib-0013] Egan, H. , Kirk, R. S. , & Sawyer, R. (1981). Pearson’s chemical analysis of foods, 8th ed (pp. 185–185). Essex: Longman Scientific and Technical.

[fsn31270-bib-0014] Falade, A. O. , & Oboh, G. (2015). Thermal oxidation induces lipid peroxidation and changes in the physicochemical properties and β‐carotene content of arachis oil. International Journal of Food Science, 2015, 1–7. 10.1155/2015/806524 PMC4745487 26904665

[fsn31270-bib-0015] FAO (1986). FAO Food and Nutrition paper manuals of food quality control food analysis: Quality, adulteration, and tests of identity. Rome, Italy: Food and Agriculture Organization of the United Nations.

[fsn31270-bib-0016] Farkas, T. (1979). Adaptation of fatty acid composition to temperature. A study on planktonic crustaceans. Comparative Biochemistry and Physiology Part B: Comparative Biochemistry, 64(1), 71–76. 10.1016/0305-0491(79)90185-8 6518757

[fsn31270-bib-0017] Faustman, C. , Specht, S. M. , Malkus, L. A. , & Kinsman, D. M. (1992). Pigment oxidation in ground veal: Influence of lipid oxidation, iron and zinc. Meat Science, 31, 351–362. 10.1016/0309-1740(92)90064-B 22059635

[fsn31270-bib-0018] Gharibzahedi, S. M. T. , & Mohammadnabi, S. (2017). Effect of novel bioactive edible coatings based on jujube gum and nettle oil‐loaded nanoemulsions on the shelf‐life of Beluga sturgeon fillets. International Journal of Biological Macromolecules, 95, 769–777. 10.1016/j.ijbiomac.2016.11.119 27919809

[fsn31270-bib-0019] Gou, J. , Choi, G. P. , & Ahn, J. (2012). Biochemical quality assessment of semi‐dried squid (*Todarodes pacificius*) treated with high hydrostatic pressure. Journal of Food Biochemistry, 36(2), 171–178. 10.1111/j.1745-4514.2010.00523.x

[fsn31270-bib-0020] Guizani, N. , Rahman, M. S. , Al‐Ruzeiqi, M. H. , Al‐Sabahi, J. N. , & Sureshchandran, S. (2014). Effects of brine concentration on lipid oxidation and fatty acids profile of hot smoked tuna (*Thunnus albacares*) stored at refrigerated temperature. Journal of Food Science and Technology, 51(3), 577–582. 10.1007/s13197-011-0528-4 24587535 PMC3931871

[fsn31270-bib-0021] Harada, H. , Tsujino, T. , Watari, Y. , Nonaka, H. , Emoto, N. , & Yokoyama, M. (2004). Oral taurine supplementation prevents fructose‐induced hypertension in rats. Heart and Vessels, 19(3), 132–136. 10.1007/s00380-003-0757-1 15168061

[fsn31270-bib-0022] Hashem, M. , & Alamri, S. (2010). Contamination of common spices in Saudi Arabia markets with potential mycotoxin‐producing fungi. Saudi Journal of Biological Sciences, 17(2), 167–175. 10.1016/j.sjbs.2010.02.011 23961074 PMC3730882

[fsn31270-bib-0023] Heo, S. J. , Park, E. J. , Lee, K. W. , & Jeon, Y. J. (2005). Antioxidant activities of enzymatic extracts from brown seaweeds. Bioresource Technology, 96(14), 1613–1623. 10.1016/j.biortech.2004.07.013 15978995

[fsn31270-bib-0024] Hong, H. , Luo, Y. , Zhou, Z. , & Shen, H. (2012). Effects of low concentration of salt and sucrose on the quality of bighead carp (Aristichthys nobilis) fillets stored at 4 °C. Food Chemistry, 133, 102–107. 10.1016/j.foodchem.2012.01.002

[fsn31270-bib-0025] Hsu, H. H. , Chuang, T. C. , Lin, H. C. , Huang, Y. R. , Lin, C. M. , Kung, H. F. , & Tsai, Y. H. (2009). Histamine content and histamine‐forming bacteria in dried milkfish (*Chanos chanos*) products. Food Chemistry, 114(3), 933–938. 10.1016/j.foodchem.2008.10.040

[fsn31270-bib-0026] Hwang, C. C. , Lin, C. M. , Kung, H. F. , Huang, Y. L. , Hwang, D. F. , Su, Y. C. , & Tsai, Y. H. (2012). Effect of salt concentrations and drying methods on the quality and formation of histamine in dried milkfish (*Chanos chanos*). Food Chemistry, 135(2), 839–844. 10.1016/j.foodchem.2012.05.035 22868167

[fsn31270-bib-0027] ICMSF (1988). Microorganisms in foods 4. Applications of the Hazard Analysis Critical Control Point (HCCP) system to ensure microbiological safety and quality. Oxford, UK: Blackwell Scientific Publications (ISBN 0‐632‐2781‐0).

[fsn31270-bib-0028] Joo, D. S. (2011). Changes in quality of salted and dried brown‐croaker product prepared with deep seawater salt. Journal of the Korean Society of Food Science and Nutrition, 40(2), 235–244. 10.3746/jkfn.2011.40.2.235

[fsn31270-bib-0029] Kim, H. S. , Seong, J. H. , Lee, Y. G. , Xie, C. L. , Choi, W. S. , Kim, S. H. , & Yoon, H. D. (2009). Effect of low molecular‐weight collagen peptide extract isolated from scales of the flathead mullet (*Mugil cephalus*) on lipid metabolism in hyperlipidemic rats. The Journal of Korean Society of Food Preservation, 16(6), 938–945.

[fsn31270-bib-0030] Kim, M. H. , Kim, M. C. , Park, J. S. , Kim, J. W. , & Lee, J. O. (2001). The antioxidative effects of the water soluble extracts of plants used as tea materials. Korean Journal of Food Science and Technology, 33(1), 12–18.

[fsn31270-bib-0031] Kim, M. M. , Rajapakse, N. , & Kim, S. K. (2009). Antiinflammatory effect of *Ishige okamurae* ethanolic extract via inhibition of NF‐κB transcription factor in RAW 264.7 cells. Phytotherapy Research, 23(5), 628–634. 10.1002/ptr.2674 19117331

[fsn31270-bib-0032] Köse, S. , Koral, S. , Özoğul, Y. , & Tufan, B. (2010). Fatty acid profile and proximate composition of Pacific mullet (Mugil so‐iuy) caught in the Black Sea. International Journal of Food Science and Technology, 45(8), 1594–1602. 10.1111/j.1365-2621.2010.02309.x|

[fsn31270-bib-0033] Kung, H. F. , Chien, L. T. , Liao, H. J. , Lin, C. S. , Liaw, E. T. , Chen, W. C. , & Tsai, Y. H. (2008). Chemical characterisation and histamine‐forming bacteria in salted mullet roe products. Food Chemistry, 110(2), 480–485. 10.1016/j.foodchem.2008.02.029 26049242

[fsn31270-bib-0034] Laemmli, V. K. (1970). Cleavage of structural proteins during the assembly of the heads of bacteriophage T4. Nature, 227(5259), 680–685. 5432063 10.1038/227680a0

[fsn31270-bib-0035] Lauzon, H. L. , Margeirsson, B. , Sveinsdóttir, K. , Guðjónsdóttir, M. , Karlsdóttir, M. G. , & Martinsdóttir, E. (2010). Overview on fish quality research. Impact of fish handling, processing, storage and logistics on fish quality deterioration (pp. 1–6). Iceland: Icelandic Food and Biotech R&D.

[fsn31270-bib-0036] Lee, I. S. , Kim, C. I. , Chae, M. H. , & Chang, C. H. (2007). Storage and acceptability of a smoked Sebastes schlegeli product. Journal of the Korean Society of Food Science and Nutrition, 36(11), 1458–1464. 10.3746/jkfn.2007.36.11.1458

[fsn31270-bib-0037] Lee, S. H. , Moon, W. S. , & Park, K. N. (2000). Antimicrobial activity of *Caesalpina sappan* L. extracts and its effect on preservation of ground meats. Journal of the Korean Society of Food Science and Nutrition, 29, 888–892.

[fsn31270-bib-0038] Lee, Y. W. , & Park, Y. H. (1985). Effect of partial freezing as a keeping freshness. 1. Changes in freshness and gel forming ability of mullet muscle during storage by partial freezing. Bulletin of the Korean Fisheries Society, 18(6), 529–537.

[fsn31270-bib-0039] Lu, J. Y. , Ma, Y. M. , Williams, C. , & Chung, R. A. (1979). Fatty and amino acid composition of salted mullet roe. Journal of Food Science, 44(3), 676–677. 10.1111/j.1365-2621.1979.tb08473.x

[fsn31270-bib-0040] Ma, Y. , Wu, X. , Zhang, Q. , Giovanni, V. , & Meng, X. (2018). Key composition optimization of meat processed protein source by vacuum freeze‐drying technology. Saudi Journal of Biological Sciences, 25(4), 724–732. 10.1016/j.sjbs.2017.09.013 29740237 PMC5936978

[fsn31270-bib-0041] Maqsood, S. , Benjakul, S. , & Shahidi, F. (2013). Emerging role of phenolic compounds as natural food additives in fish and fish products. Critical Reviews in Food Science and Nutrition, 53(2), 162–179. 10.1080/10408398.2010.518775 23072531

[fsn31270-bib-0042] Marais, J. F. K. , & Erasmus, T. (1977). Body composition of *Mugil cephalus, Liza dumerili, Liza richardsoni* and *Liza tricuspidens* (Teleostei: Mugilidae) caught in the Swartkops estuary. Aquaculture, 10(1), 75–86. 10.1016/0044-8486(77)90034-5

[fsn31270-bib-0043] Matsubara, K. , Matsuura, Y. , Hori, K. , & Miyazawa, K. (2000). An anticoagulant proteoglycan from the marine green algae, Codiumpugniformis. Journal of Applied Psychology, 12(1), 9–14. 10.1023/A:1008174115350

[fsn31270-bib-0044] Matsushima, Y. , Sekine, T. , Kondo, Y. , Sakurai, T. , Kameo, K. , Tachibana, M. , & Murakami, S. (2003). Effects of taurine on serum cholesterol levels and development of atherosclerosis in spontaneously hyperlipidaemic mice. Clinical and Experimental Pharmacology and Physiology, 30(4), 295–299. 10.1046/j.1440-1681.2003.03828.x 12680850

[fsn31270-bib-0045] Muraleedharan, V. , Antony, K. P. , Perigreen, P. A. , & Gopakumar, V. (1996). Utilization of unconventional fish resources for surimi preparation. Proceeding of the second workshop on scientific results of FORV SAGAR Sampada, Dept. of Ocean Development (pp. 539–543). New Delhi. India.

[fsn31270-bib-0046] Noël, L. , Chafey, C. , Testu, C. , Pinte, J. , Velge, P. , & Guerin, T. (2011). Contamination levels of lead, cadmium and mercury in imported and domestic lobsters and large crab species consumed in France: Differences between white and brown meat. Journal of Food Composition and Analysis, 24(3), 368–375. 10.1016/j.jfca.2010.08.011

[fsn31270-bib-0047] Nooralabettu, K. P. (2008). Effect of sun drying and artificial drying of fresh, salted Bombay duck (*Harpodon neherius*) on the physical characteristics of the product. Journal of Aquatic Food Product Technology, 17, 99–116. 10.1080/10498850801936994

[fsn31270-bib-0048] Norouzi, M. , & Bagheri, M. (2015). The chemical composition of golden grey mullet Liza aurata in southern Caspian Sea during sexual rest and sexual ripeness. AACL Bioflux, 8(4), 517–525.

[fsn31270-bib-0049] Northrop, J. H. (1926). A convenient method for the formal titration. The Journal of General Physiology, 9, 767–769. 19872291 10.1085/jgp.9.6.767 PMC2140909

[fsn31270-bib-0050] Osman, H. , Suriah, A. R. , & Law, E. C. (2001). Fatty acid composition and cholesterol content of selected marine fish in Malaysian waters. Food Chemistry, 73(1), 55–60. 10.1016/S0308-8146(00)00277-6

[fsn31270-bib-0051] Pearson, D. (1970). The Chemical Analysis of Food, 6th ed London, UK: J.A Church Hill.

[fsn31270-bib-0052] Pollero, R. J. , Ré, M. E. , & Brenner, R. R. (1979). Seasonal changes of the lipids of the mollusc *Chlamys tehuelcha* . Comparative Biochemistry and Physiology Part A: Physiology, 64(2), 257–263. 10.1016/0300-9629(79)90658-3

[fsn31270-bib-0053] Qiu, L. , Zhang, M. , Tang, J. , Adhikari, B. , & Cao, P. (2019). Innovative technologies for producing and preserving intermediate moisture foods: A review. Food Research International, 116, 90–102. 10.1016/j.foodres.2018.12.055 30717022

[fsn31270-bib-0054] Qwele, K. , Hugo, A. , Oyedemi, S. O. , Moyo, B. , Masika, P. J. , & Muchenje, V. (2013). Chemical composition, fatty acid content and antioxidant potential of meat from goats supplemented with Moringa (Moringa oleifera) leaves, sunflower cake and grass hay. Meat Science, 93(3), 455–462. 10.1016/j.meatsci.2012.11.009 23273450

[fsn31270-bib-0055] Rosa, A. , Scano, P. , Melis, M. P. , Deiana, M. , Atzeri, A. , & Dessi, M. A. (2009). Oxidative stability of lipid components of mullet (*Mugil cephalus*) roe and its product “bottarga”. Food Chemistry, 115(3), 891–896. 10.1016/j.foodchem.2009.01.002

[fsn31270-bib-0056] Ruxton, C. H. , Reed, S. C. , Simpson, M. J. , & Millington, K. J. (2004). The health benefits of omega‐3 polyunsaturated fatty acids: A review of the evidence. Journal of Human Nutrition and Dietetics, 17(5), 449–459. 10.1111/j.1365-277X.2004.00552.x 15357699

[fsn31270-bib-0057] Schormuller, J . (1968). Handbuch der Lebensmittel Chemie, Band 3/2 Teil, Trierische Lebensmittel Eier, Fleisch, Fisch, Buttermilch (pp. 872–878). Berlin, Germany: Springer‐Verlag.

[fsn31270-bib-0058] Seki, N , & Watanabe, T . (1984). Connectin content and its postmortem changes in fish muscle. Journal of Biochemistry, 95(4), 1161–1167. 10.1093/oxfordjournals.jbchem.a134705 6746595

[fsn31270-bib-0059] Siriskar, D. A. , Khedkar, G. D. , & Lior, D. (2013). Production of salted and pressed anchovies (*stolephorus sp*.) and it's quality evaluation during storage. Journal of Food Science and Technology, 50(6), 1172–1178. 10.1007/s13197-011-0450-9 24426031 PMC3791226

[fsn31270-bib-0060] Song, H. N. , Lee, D. G. , Han, S. W. , Yoon, H. K. , & Hwang, I. K. (2005). Quality changes of salted and semi‐dried mackerel fillets by UV treatment during refrigerated storage. Korean Journal of Food and Cookery Science, 21(5), 662–668.

[fsn31270-bib-0061] Taşkaya, L. , & Yaşar, E. (2018). Determination of some quality properties of "hamsi kaygana" prepared with different additives. Food Science & Nutrition, 6(2), 483–491. 10.1002/fsn3.578 29564116 PMC5849902

[fsn31270-bib-0062] Thomson, J. M. (1966). The grey mullets. Oceanography and Marine Biology‐An Annual Review, 4, 301–355.

[fsn31270-bib-0063] Witte, V. C. , Krause, G. F. , & Baile, M. E. (1970). A new extraction method for determining 2‐thiobarbituric acid values of pork and beef during storage. Journal of Food Science, 35(5), 582–585. 10.1111/j.1365-2621.1970.tb04815.x

[fsn31270-bib-0064] Yang, S. T. (1997). Preparation of seasoned and semi‐dried horse mackerel by cold air drying and quality of its product during partially frozen storage. Food Science and Technology, 29(5), 925–931.

[fsn31270-bib-0065] Yin, X. F. , Kim, K. B. , Noh, J. S. , & Choi, S. K. (2013). Quality characteristics of cod bone stock containing various amount of tomatoes. The Korean Journal of Culinary Research, 19(4), 231–242. 10.20878/cshr.2013.19.4.016

[fsn31270-bib-0066] You, B. J. (1997). Changes of salmon meat texture during semi‐ drying process. Korean Journal of Fisheries and Aquatic Sciences, 30(2), 264–270.

[fsn31270-bib-0067] You, S. H. , Shin, K. E. , Choi, S. K. , & Seo, Y. W. (2013). Quality characteristics of mussel stock with different heating times. The Korean Journal of Culinary Research, 19(3), 209–217. 10.20878/cshr.2013.19.3.015015015

